# Dendritic cells overcome Cre/Lox induced gene deficiency by siphoning cytosolic material from surrounding cells

**DOI:** 10.1016/j.isci.2024.109119

**Published:** 2024-02-06

**Authors:** Christopher H. Herbst, Aurélie Bouteau, Evelin J. Menykő, Zhen Qin, Ervin Gyenge, Qingtai Su, Vincent Cooper, Neil A. Mabbott, Botond Z. Igyártó

**Affiliations:** 1 Department of Microbiology and Immunology, Thomas Jefferson University, Philadelphia, PA 19107, USA; 2 OncoNano Medicine, Inc, Southlake, TX 76092, USA; 3 The Roslin Institute & Royal (Dick) School of Veterinary Studies, University of Edinburgh, Edinburgh, UK

**Keywords:** Cell biology, Components of the immune system, Immunology

## Abstract

In a previous report, keratinocytes were shown to share their gene expression profile with surrounding Langerhans cells (LCs), influencing LC biology. Here, we investigated whether transferred material could substitute for lost gene products in cells subjected to Cre/Lox conditional gene deletion. We found that in human Langerin-Cre mice, epidermal LCs and CD11b+CD103+ mesenteric DCs overcome gene deletion if the deleted gene was expressed by neighboring cells. The mechanism of material transfer differed from traditional antigen uptake routes, relying on calcium and PI3K, being susceptible to polyguanylic acid inhibition, and remaining unaffected by inflammation. Termed intracellular monitoring, this process was specific to DCs, occurring in all murine DC subsets tested and human monocyte-derived DCs. The transferred material was presented on MHC-I and MHC-II, suggesting a role in regulating immune responses.

## Highlights

• Langerhans cells (LCs) and dendritic cells (DCs) overcome Cre/Lox induced gene deletions
• LCs and DCs acquire material via a novel mechanism termed intracellular monitoring
• Intracellular monitoring is specific to DCs and LCs, and unaffected by inflammatory stimuli
• Acquired antigen is processed, presented, and cross-presented on MHC-I/II

## Graphical abstract

**Figure:**
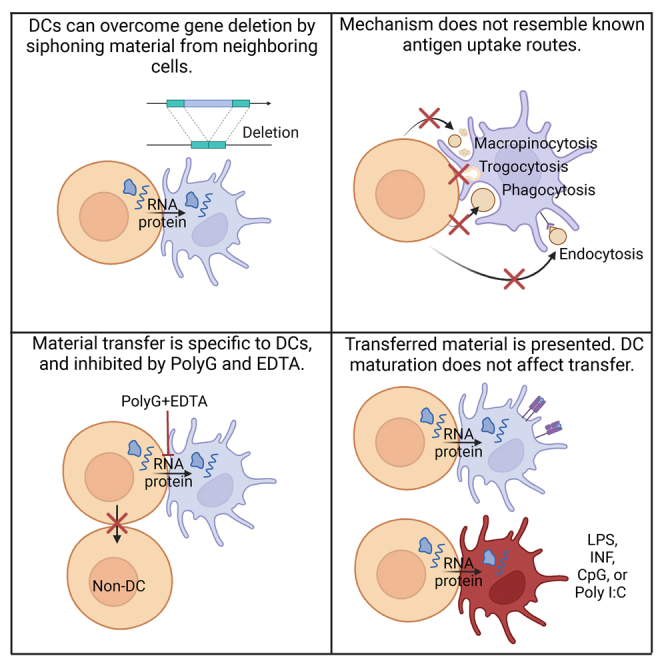

## Introduction

Dendritic cells (DCs) are the critical link between innate and adaptive immunity. At steady state, DCs in peripheral tissue scavenge their surroundings for antigens in the form of apoptotic bodies, cell debris, and extracellular vesicles. If they encounter molecules that ligate pattern recognition receptors, they become activated, upregulate costimulatory molecules and MHC-II on their surface, and migrate to lymph nodes to present antigens to adaptive immune cells.^1^^,^^2^ When cells die or release material into the extracellular environment, DCs are thought to acquire it through multiple endocytic processes, including receptor-mediated endocytosis, phagocytosis, and macropinocytosis, which they conduct at high rates.^3^ However, more recent literature has challenged this notion of DCs as scavengers by showing that DCs acquire and cross-present antigens equally well from live cells as they do apoptotic.^4^^,^^5^ Further, compared to DCs that acquire antigen from apoptotic cells, DCs acquiring antigen from live cells generate larger CD8^+^ T cell responses and increased protection from lethal tumor challenge when injected *in vivo*.^6^ Separating DCs from live donor cells with a 0.45 μm pore size transmembrane insert prevents cross-presentation, indicating live cell contact-dependent antigen uptake is critical for inducing an adaptive response.^4^

Importantly, by acquiring material from live cells, DCs can interact with a variety of molecules that are not usually present in scavenged material. For example, mRNA is degraded as cells go through apoptosis,^7^ and the total volume of material that can be transferred through extracellular vesicles is restricted by their small size. By circumventing these restrictions, DCs can contain large, functionally relevant quantities of RNA and protein from their surroundings. The immunological impact of such transfer has been observed in numerous contexts. Antigen acquired by metallophilic marginal zone macrophages in the spleen is actively transferred to DCs, which can promote or suppress adaptive immunity depending on context.^8^ A similar transfer is seen between CXCR1+ macrophages and CD103+ DCs in the context of oral tolerance,^9^ and macrophages are known to siphon cytosolic material from stem cells during quality control checks.^10^ Our lab found that epidermal Langerhans cells (LCs) contain certain KC-derived mRNA at nearly 50% of the level present in KCs themselves.^11^ Aside from its biological importance, high volume transfer of material from one cell type to another is of concern for researchers utilizing conditional knockout animals.

Cre/Lox animal models are a standard tool for deleting genetic regions in specific cell types. This is accomplished by placing the expression of the bacterial recombinase Cre under the control of a cell type-specific promoter. When expressed, Cre will act on two short *LoxP* sequences that have been inserted into the gene of interest, resulting in cell type-specific gene disruption. However, if DCs can acquire a large enough quantity of material from neighboring cells, the efficacy of Cre/Lox models may be undermined. Our lab has already shown that DCs can acquire Cre expressed by neighboring cells, potentially resulting in off-target effects,^11^ but it remains to be seen whether the material transfer can overcome DC-specific gene deletion. If so, many DC-specific conditional knockout models may be non-functional at the protein level despite successful genetic recombination. Considering the broad usage of such models, further investigation of this concern is warranted.

Of equal intrigue is how DCs are acquiring this material from their neighbors in the first place. Mechanistically, contact-dependent material transfer between live cells can occur through trogocytosis, tunneling nanotubes (TNTs), or gap junctions. Trogocytosis, an active “nibbling” process that results in the transfer of surface molecules and membrane fragments,^12^ is routinely used by DCs to acquire peptide:MHC from neighboring cell membranes in a process called cross-dressing, which is important for T cell activation in response to viral infection.^13^ It is also suspected that thymic DCs use trogocytosis to acquire antigen expressed by medullary thymic epithelial cells to help maintain central tolerance.^14^^,^^15^ TNTs, originally described in 2004,^16^ are thin, F-actin containing protrusions that enable open-ended connections between cells at a distance. Genes required for their formation are highly expressed in many DCs,^17^ and endosomes, viral antigens, and peptide-MHC complexes transfer through them during type one immune response.^18^ Gap junctions enable bidirectional exchange of molecules under 1 kD, including ions, metabolites, small RNAs, and antigenic peptides.^19^ In some form, all of these mechanisms enable antigen uptake from live cells. However, considering that trogocytosis predominantly facilitates membrane transfer, *in vivo* evidence of RNA transfer between heterogeneous cell types by TNTs is scarce, and large RNA molecules do not pass through gap junctions, it is difficult to account for the large quantity of material transfer to LCs with them alone. Thus, we also sought to investigate how DCs acquire material from their neighbors.

Herein, we show that LCs and CD11b+CD103+ mesenteric lymph node DCs are able to overcome Cre/Lox induced gene deficiency by siphoning RNA and protein from neighboring cells. This ability is mostly exclusive to DCs, shared among all DC subsets tested, and conserved in human monocyte-derived DCs. *In vitro*, inhibitors targeting conventional means of antigen uptake fail to prevent siphoning. Instead, DCs use a contact-dependent mechanism which we term *intracellular monitoring* (ICM)*.* ICM is dependent on extra and intracellular calcium, can be blocked by polyguanylic acid, and is not altered by inflammation. Material siphoned through this mechanism is presented on MHC-I and MHC-II.

## Results

### DCs can overcome gene deficiency

**Figure 1:**
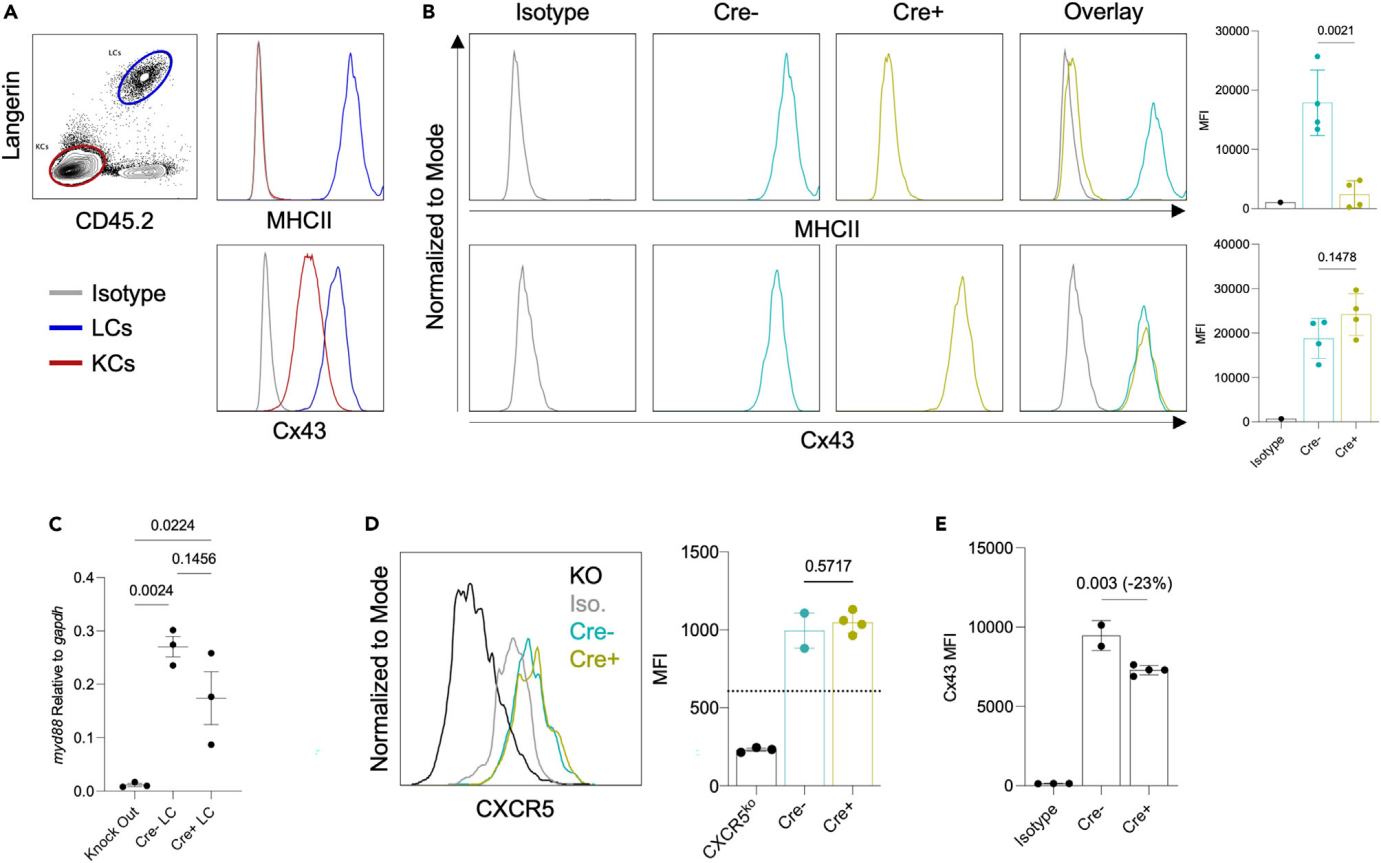
DCs can overcome gene deficiency (A) Gating strategy for identifying keratinocytes and Langerhans cells in an epidermal cell suspension. Epidermal cell suspension from a WT mouse was stained for MHC-II (top) or Cx43 (bottom) or corresponding isotype controls. KCs: keratinocytes, LCs: Langerhans cells. Isotype control signal shown for keratinocytes. Representative flow plots. (B) Expression of the indicated proteins by epidermal Langerhans cells derived from MHC-II or Cx43 hLangCre conditional knockout mice. Representative flow plots and summary graphs. Dots represent individual mice from one of three independent experiments. (C) *myd88* mRNA level relative to housekeeping gene *gapdh* in Cre- or Cre+ LCs sorted from hLangCre MyD88^f/f^ or global myd88 knockout epidermis, quantified by qPCR. Dots represent individual mice from one out of three independent experiments. MyD88 KO mice included in one of three repeat experiments. (D) Representative CXCR5 flow staining of splenic DCs from a global CXCR5 knockout mouse (KO), or skin draining lymph node migratory LCs from either Cre- (blue) or Cre+ (green) hLangCre CXCR5^f/f^ mice, or isotype control staining of skin draining lymph node migratory LCs from Cre-hLangCre CXCR5^f/f^ mice, and summary graphs. Dotted line represents isotype control. Dots represent individual mice from one of two independent experiments. (E) Cx43 flow staining of CD11b+CD103+ mesenteric lymph node DCs derived from either Cre- or Cre+ hLangCre Cx43^f/f^ mice, or isotype control staining of Cre-hLangCre Cx43^f/f^ mesenteric lymph node DCs. Dots represent individual mice from one of three independent experiments. Data are represented as mean ± SD.

We previously reported that keratinocytes (KCs) share their gene expression profile with the surrounding LCs, affecting LC biology.^11^ Here, we tested whether this material transfer could overcome gene deficiencies in LCs. First, we selected two proteins: Cx43 (gap junction protein connexin 43) and MHC-II. The rationale behind these proteins was that our previous ATAC-seq data showed that the gene coding for Cx43 was open for transcription in KCs but not LCs, while the gene coding for MHC-II was open for transcription in LCs but not KCs.^11^ Thus, we expected that WT LCs would contain MHC-II, but not Cx43, unless the LCs acquired it from KCs. Flow cytometry analyses of the WT epidermis showed that MHC-II is not detected in KCs, but both Cx43 and MHC-II proteins are present in LCs (Figure 1A). The specificity of the anti-Cx43 antibody used here was validated on Cx43 KO cells (clone: CX-1B1; advanced verification by ThermoFisher). Furthermore, ImmGen microarray data corroborated our findings and showed high Cx43 (*gja1*) RNA levels in epidermal LCs (Figure S1A). Thus, these data confirmed our previous findings that LCs can acquire proteins and RNA from the surrounding cells.^11^ To rule out the possibility that LCs might still express the gene coding for Cx43 and directly test whether material transfer can overcome conditional gene deletion, we assessed target gene protein and mRNA levels in the previously characterized hLangCre-MHC-II^f/f^ mice,^20^ and *de novo* in-house generated hLangCre-Gja-1^f/f^ (Cx43)^21^ mice. The specific genomic recombination of the Cx43 locus in LCs was confirmed using PCR on sorted cells (Figure S1B). Flow cytometry staining for MHC-II and Cx43 revealed that while epidermal LCs from Cre+ mice did not have MHC-II, they did have similar levels of Cx43 protein (Figure 1B) and RNA (Figure S1C) as their Cre-counterparts. These findings, therefore, support that LCs can overcome conditional gene deletion if neighboring cells express the missing gene.

To increase rigor, we further tested the system using two other floxed mouse strains from our hLangCre colony: the hLangCre-MyD88^f/f^ and the hLangCre-CXCR5^f/f^ mice. The hLangCre-MyD88^f/f^ mice were previously published,^22^ while the hLangCre-CXCR5^f/f^ mice were generated for a different project in-house by breeding the hLangCre mice to CXCR5^f/f^ mice.^23^ Both mouse strains showed successful gene recombination in LCs (Figures S1D and S1E). Similar to MHC-II and Cx43 protein staining presented above, we sought to assess MyD88 and CXCR5 protein expression levels by flow cytometry. However, the only anti-MyD88 antibody that has been previously reported to generate a positive signal in flow cytometry (MyD88 clone 4D6, Novus Biologicals NBP2-27369) showed WT-level MyD88 staining in global MyD88 KO mice purchased from Jax (Figure S1F), rendering it unusable for our purposes. The anti-CXCR5 antibody, whose specificity we confirmed on B cells from WT and CXCR5 KO mice (Figure S1G), did not reveal any significant staining in WT epidermal LCs and KCs (Figure S1G). Therefore, to determine whether deletion of MyD88 in LCs can be overcome, we used qPCR on flow-sorted cells. We found that the MyD88 transcripts levels in LCs were not significantly different between the Cre- and Cre+ mice but were well above global MyD88 knockout levels (Figure 1C), and that the KCs had comparable MyD88 to LCs (Figure S1H). Thus, these data further support that LCs can overcome gene deficiency if localized in a transcript-sufficient niche. Since the epidermal LCs migrate from a CXCR5 deficient epidermal environment to the lymph node, which can be considered as a high CXCR5 niche, we hypothesized that CXCR5 deficient epidermal LCs would become CXCR5+ in the lymph nodes. Indeed, flow cytometry on LCs from the skin-draining lymph nodes of hLangCre-CXCR5^f/f^ mice showed similar levels of positive CXCR5 staining for Cre- and Cre+ mice (Figure 1D). These findings are in concordance with the previously published observation that LCs in the hLangCre-MHC-II^f/f^ mice acquire MHC-II positivity in the lymph node and induce T cell responses,^24^ and further support our hypothesis that the local environment will dictate whether LCs can overcome gene deficiency.

Though LCs possess DC-like properties, they are derived from monocytes and considered macrophages with DC characteristics.^25^ To determine whether bona fide DCs can also overcome gene deficiencies, we took advantage of the fact that the human langerin promoter in the hLangCre mice drives Cre expression in the CD11b+CD103+ double-positive DCs (Figure S1I) in the lamina propria and the mesenteric lymph nodes.^26^ Therefore, we assessed the expression of Cx43 in these DCs. As with the skin and skin-draining lymph nodes, we found that double-positive DCs remained Cx43 sufficient in the mesenteric lymph nodes (Figure 1E). Thus, these data support that the ability to overcome gene deficiencies is likely universal among DCs.

### Intracellular material acquisition from surrounding cells is specific to DCs and universal among all DC subsets tested

**Figure 2:**
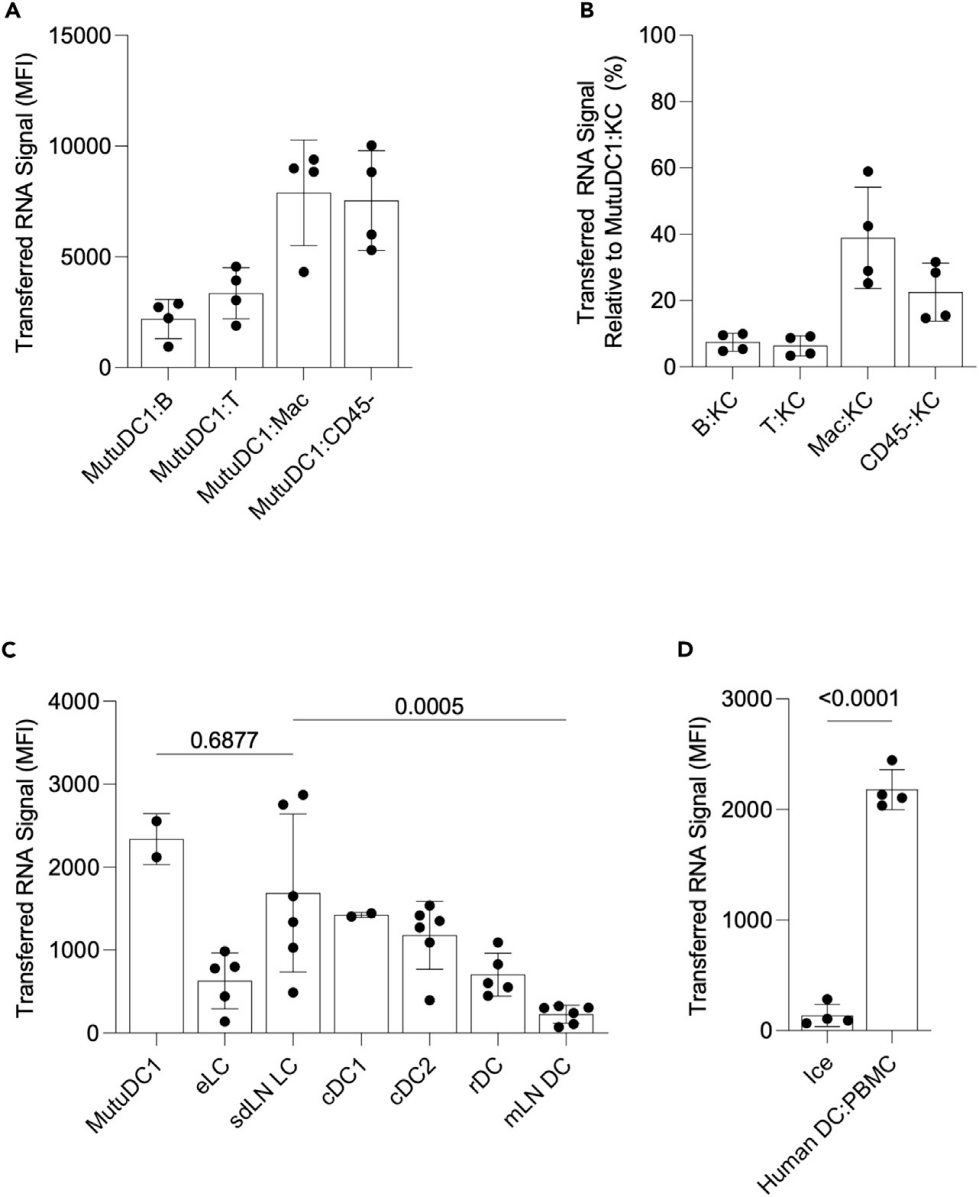
Intracellular material acquisition from surrounding cells is specific to DCs and universal among all DC subsets tested (A) Splenic B cells (B), T cells (T), peritoneal macrophages (Mac), or dermal CD45^−^cells (CD45^−^) were sorted from wild type C57BL/6 mice, labeled with SYTO62 and co-cultured with MutuDC1 cells for 45 min. Dots represent individual mice. Data combined from two experiments. (B) Sorted cells were labeled with CFSE and co-cultured for 45 min with SYTO62 labeled COCA keratinocytes (KC). Points represent individual mice. Data normalized and combined from two experiments. (C) Epidermal Langerhans cells (eLC), skin draining lymph node migratory Langerhans cells (sdLN LC), sdLN cDC1, sdLN cDC2, resident DCs (rDC), and mesenteric lymph node migratory DCs (mLN DC) were sorted from hLangCre-YFP^f/f^ mice, labeled with CFSE, and co-cultured with RNA labeled COCA KCs for 45 min. Dots represent individual mice. Data pooled from three experiments. (D) moDCs were differentiated from human CD14^+^ monocytes and labeled with CFSE, then co-cultured with RNA labeled PBMCs on ice or 37°C for 45 min. Dots represent individual replicates. One representative experiment of two is shown. Data are represented as mean ± SD.

The data above suggest that DCs can acquire cytosolic material from different cell types. To determine which cell types DCs monitor, we used a modified *in vitro* co-culture system that we developed to image RNA acquisition by LCs from epidermal KCs.^11^ Briefly, we co-cultured GFP-expressing MutuDC1 cells (DC cell line with cDC1 phenotypical and functional characteristics)^27^ with SYTO62-labeled (nucleic acid dye) B cells, T cells, peritoneal macrophages, or dermal CD45^−^cells sorted from adult wild-type naive C57BL/6 mice (Figures S2A and S2B). Results show that while DCs acquire RNA from all cell types tested, they most efficiently siphon from macrophages and non-hematopoietic CD45^−^stromal cells (Figure 2A). Thus, DCs can monitor both hematopoietic and non-hematopoietic cells.

To better understand the role of intracellular material acquisition, we sought to determine if it is exclusive to DCs, or a property of many cell types. To do that, we co-cultured the sorted T cells, B cells, macrophages and CD45^−^stromal cells with RNA labeled keratinocytes from the murine cell line COCA.^28^ We observed that RNA transfer ranged from significantly less efficient (macrophage and stromal cells) to almost entirely absent (T and B cells) when using cell types other than DCs as recipients (Figure 2B). In addition, we observe minimal RNA transfer when MutuDC1 cells are used as donors, indicating that transfer is mostly unidirectional in favor of DCs (Figure S2C). These findings support that intracellular monitoring is a unique DC property.

We previously reported that DC subsets harbor mRNAs specific to their tissue of residence.^11^ To bring experimental evidence that other DC subsets can acquire RNA from the surrounding cells, we sorted DC subsets from single-cell suspensions generated from the epidermis, skin-draining lymph nodes, and mesenteric lymph nodes of adult naive mice (Figure S2D) and co-cultured them with RNA labeled COCA KCs. We found that all DC subsets of the epidermis and skin-draining lymph nodes were able to acquire RNA from KCs to varying degrees (Figure 2C), while mesenteric CD11b+CD103+ DCs acquired some, but significantly less RNA—aligning well with their lesser ability to overcome CX43 knockout relative to eLCs. Therefore, these data support that the material acquisition from the target cells by DCs is widespread and not limited to LCs or MutuDC1s.

We previously showed that human LCs, similar to mouse LCs, contain KC-derived keratins.^11^ To provide support that intracellular material acquisition is conserved in humans, we determined whether closely representative human monocyte-derived DCs (moDCs) can acquire RNA from other cells. We differentiated DCs from CD14^+^ blood monocytes (Figure S2E) and incubated them with autologous PBMCs labeled with RNA dye at 37°C or on ice. We found that the moDCs were efficient in acquiring RNA from the autologous PBMCs at 37°C (Figures 2D and S2F). Thus, these data support that intracellular material acquisition by DCs exists in humans.

### Intracellular material transfer is contact-dependent, but independent of known antigen acquisition pathways

**Figure 3:**
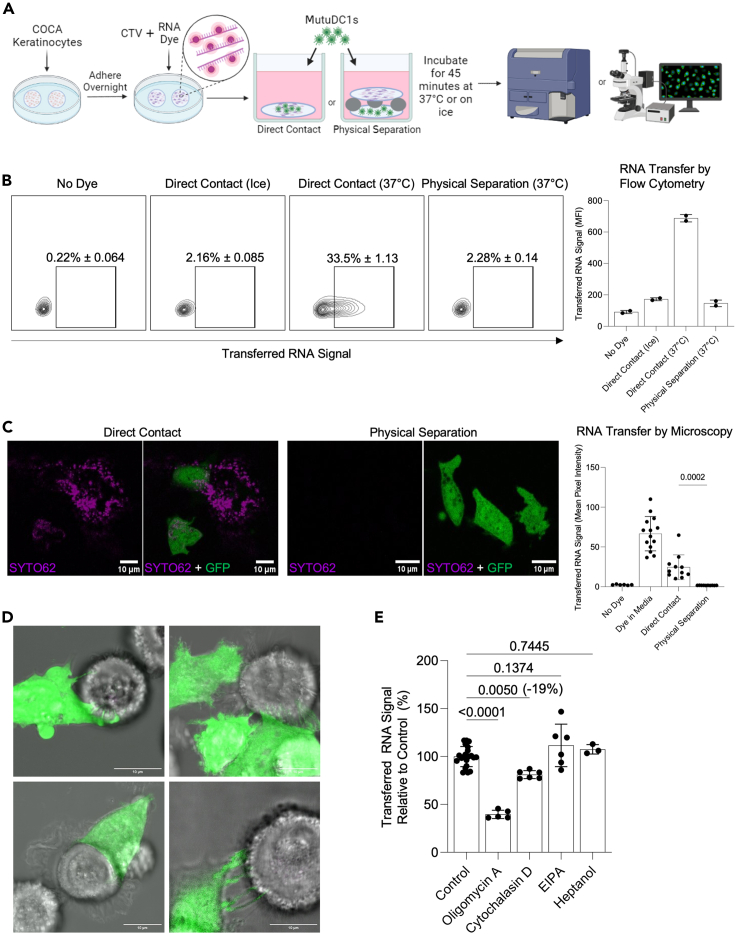
Dendritic cells siphon RNA from neighboring cells through a contact dependent mechanism that does not resemble conventional means of antigen uptake (A) Outline of experiment to measure RNA and protein transfer to MutuDC1s with or without direct contact. (B) Flow cytometric analysis of SYTO62 RNA signal measured in MutuDC1s after 45 min incubation with RNA labeled COCA KCs. Results are from a single experiment. (C) Representative images and quantification of SYTO62 signal contained within MutuDC1s after keratinocyte:DC co-cultures. Mean pixel intensity of far-red channel (SYTO62) was calculated within the area occupied by GFP+ MutuDC1s on a per cell basis. Images acquired with a Nikon A1R confocal microscope using a Plan Fluor 40× Oil objective. Dots represent individual cells. Results are from a single experiment. (D) MutuDC1s (green) interacting with COCA KCs. Max projections of z stack images taken on a Nikon A1R confocal microscope using a Plan Fluor 40× Oil objective plus 10x scanner zoom. (E) Transfer of SYTO62 labeled RNA to MutuDC1s from keratinocytes relative to vehicle controls in the presence of an ATPsynthase inhibitor (1 μM Oligomycin A), an inhibitor of F-actin formation (8 μM Cytochalasin D), a macropinocytosis inhibitor (32 μM 5-(N-Ethyl-N-isopropyl) amiloride (EIPA), and a gap junction inhibitor (5 mM 1-Heptanol). Data normalized and pooled from three experiments. Data are represented as mean ± SD.

We previously observed that LCs separated from KCs using 0.4 μm Transwell membrane are unable to acquire detectable levels of KC-derived RNA.^11^ These data suggest that free RNA, exosomes, other forms of extracellular vesicles and/or cell debris that could cross the membrane might not play a significant role in RNA transfer from KCs to LCs. However, some RNA containing microvesicles are larger than 0.4 μm,^29^ and the Transwell membrane may nonspecifically bind some vesicles and therefore hinder their access to LCs. To overcome these caveats and confirm the requirement for physical interaction for RNA transfer from KCs to DCs, we adapted an *in vitro* co-culture system^30^ where donor cells (KCs) are suspended above recipient cells (DCs) (Figure 3A). In this system, the two cell types are facing each other and are only separated by a thin layer (1.5 mm) of cell culture media. This setup provides DCs unobstructed access to KC-derived exosomes, vesicles, cell debris, and apoptotic cells, while still preventing direct contact to live adherent cells. For our system, MutuDC1 cells^27^ were seeded at the bottom of a 48-well plate and murine COCA keratinocytes were grown on round coverslips, labeled with the nucleic acid dye SYTO62 and protein dye cell trace violet (CTV), and suspended above the DCs using silicone O-rings throughout the entire culture period (“physical separation”) (Figures 3A and S3A). Physical separation again prevented transfer of RNA from KCs to DCs as measured by flow cytometry and confocal microscopy, whereas DCs that had direct physical contact with the KCs at 37°C, but not on ice, acquired KC-derived RNA (Figures 3B, 3C, and S3). Time-lapse imaging shows RNA and protein signal intensity increasing within DCs over time during direct contact (Figure S3B). Imaging also revealed that only DCs in direct contact with donor cells acquired RNA (Video S1). Together, these data strongly support that released cell vesicles, debris, and apoptotic bodies do not play a significant role in RNA transfer, and they further underpin the need for physical contact for RNA and protein transfer.

Video S1. Only DCs in direct contact with donor cells acquire nucleic acids

To gain insight into the physical interaction and mechanism that allow DCs to acquire cytosolic material from other cells, we co-cultured COCA keratinocytes with MutuDC1 cells and took confocal images and time-lapse videos of these cells interacting with one another. The physical interaction between the DCs and KCs was diverse in nature, ranging from superficial-looking touching/screening all the way to DC dendrites pressing into the KC plasma membrane (Figure 3D and Videos S2, S3, S4, and S5). Occasionally, DCs formed ring structures when contacting KCs, resulting in RNA containing vesicles. (Figure S3C and Video S3).

Video S2. Extended interactions between DCs and donor cells

Video S3. Formation of ring structures at DC:donor cell interface

Video S4. DCs interacting with CTV labeled donor cells.

Video S5. Extended dendrites sustain contact with donor cells

Having established physical interaction as a requirement for material transfer, we next tested whether previously described, standard routes of antigen acquisition, such as phagocytosis, tunneling nanotubes, macropinocytosis, and gap junctions are involved in cytosolic material acquisition by DCs. To do this, we measured RNA transfer by flow cytometry from RNA labeled COCA KCs to MutuDC1 cells during co-culture under different conditions. Transfer was significantly inhibited if they were treated with the ATP synthase inhibitor Oligomycin A,^31^ demonstrating that the process is energy-intensive, rather than a passive transfer (Figures 3E and S3D). It was recently established that tunneling nanotubes (TNTs) enable significant RNA transfer between stationary cells,^30^ and we have observed structures resembling TNTs between DCs in some of our long-term (more than 45 min) co-cultures (Figure S3E). TNTs require intact F-actin,^32^ and their formation can be inhibited with low concentrations (50 nM) of cytochalasin D, an F-actin inhibitor. Phagocytosis of detached cells or cell debris was not prevented in our physical separation system, so phagocytosis is unlikely to be contributing substantially to material transfer, however, at micromolar concentrations cytochalasin D also inhibits phagocytosis,^33^^,^^34^ so its use can further exclude phagocytosis as the dominant mechanism of transfer. Indeed, while cytochalasin D was highly effective at disrupting F-actin (Figure S3F) and inhibiting the uptake of 2 μm beads (Figure S3G), we only observed a minor inhibition (19%) of RNA transfer (Figure 3E). Increasing doses of cytochalasin D up to 100 μM did not further inhibit transfer (Figure S3H). We next sought to evaluate the contribution of macropinocytosis. 5-(N-ethyl-N-isopropyl)-Amiloride (EIPA), a Na^+^ channel inhibitor known to block macropinocytosis,^35^ did not inhibit RNA acquisition (Figure 3E), but did block fluorescent dextran uptake by DCs (Figure S3I), which is mediated partially by macropinocytosis.^36^ The gap junction inhibitor 1-heptanol^9^ also failed to inhibit RNA acquisition (Figure 3E), but did significantly reduce transfer of Calcein dye between COCA KCs (Figure S3J), which is partially mediated by gap junctions.^37^ Our findings were not limited to RNA acquisition from KCs by MutuDC1s. We observed roughly similar responses with MutuDC1s or primary splenic DCs combined with an unrelated cancer cell line, B16 (Figures S3K and S3L). Therefore, known mechanisms of material uptake such as macropinocytosis, TNTs, phagocytosis, and gap junctions do not appear to play a major role in RNA transfer.

### DCs acquire cytosolic material from other cells through a mechanism dependent on calcium and PolyG-blockable receptors

**Figure 4:**
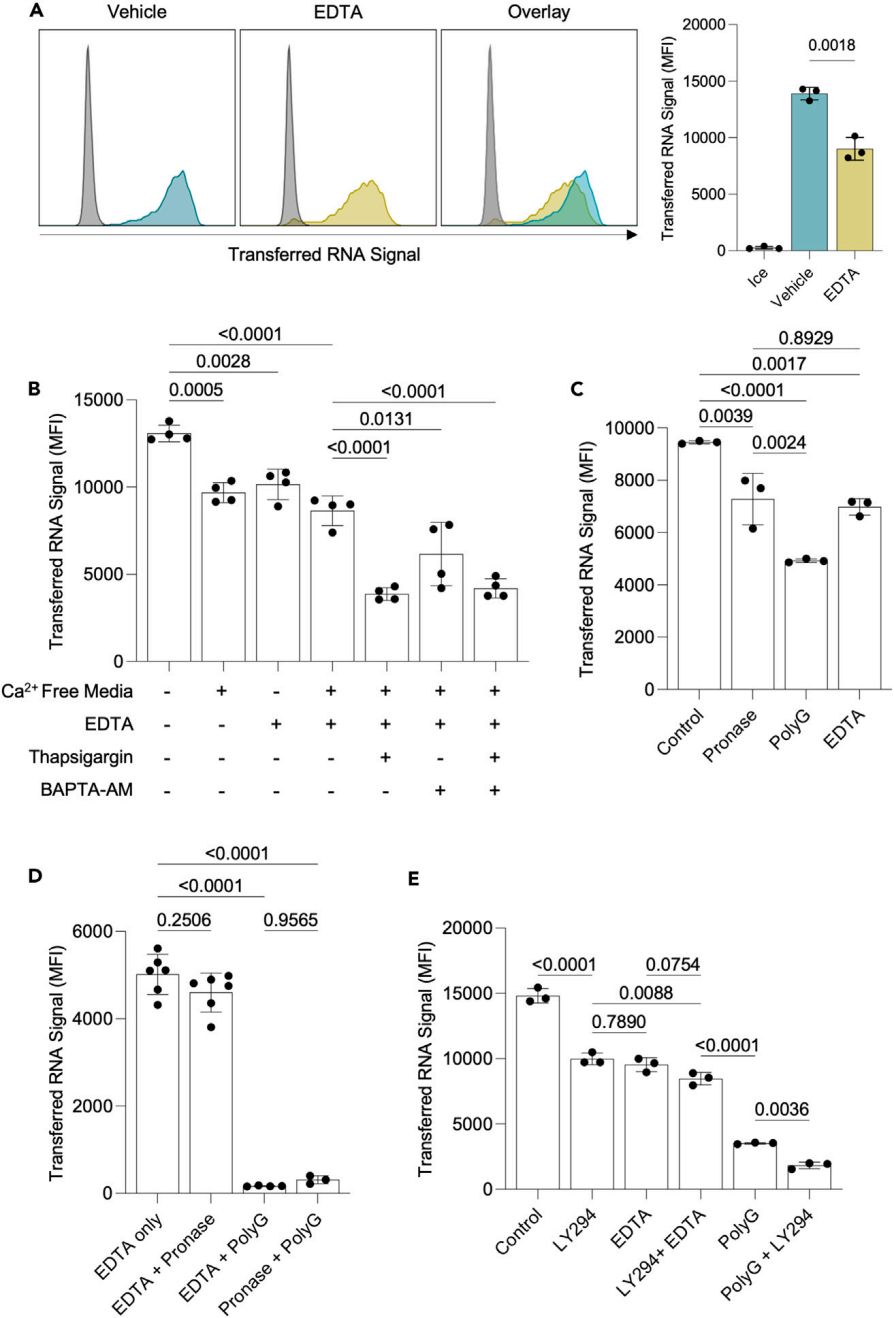
RNA transfer is dependent on calcium and can be partially blocked with the scavenger receptor inhibitor Polyguanylic acid (A) Representative histograms of MutuDC1 cells after incubation in direct contact with RNA labeled COCA keratinocytes in the presence or absence of 5 mM EDTA. (B) RNA signal in MutuDC1 cells after incubation with RNA labeled B16 cells. MutuDC1s were treated with thapsigargin (2 μM), BAPTA-AM (50 μM), or both for 30 min on ice prior to co-culture in media containing Ca^2+^, Ca^2+^ free media, or 5 mM EDTA. (C) RNA dye signal relative to control measured in MutuDC1 cells after incubation with RNA labeled B16 cells. MutuDC1s treated with 32 μg/mL Pronase or co-cultured with B16 cells in the presence or absence of 500 μg/mL PolyG, or 5 mM EDTA as indicated. (D) RNA dye signal relative to control measured in MutuDC1 cells after incubation with RNA labeled B16 cells. MutuDC1s treated with 32 μg/mL Pronase or co-cultured with B16 cells in the presence or absence of 500 μg/mL PolyG, or 5 mM EDTA as indicated. (E) RNA dye signal relative to control measured in MutuDC1 cells after incubation with RNA labeled B16 cells. MutuDC1s treated for 30 min with 50 μM LY294002, 5 mM EDTA, or 500 μg/mL PolyG as indicated. All experiments repeated at least three times. Representative results from a single experiment shown. Data are represented as mean ± SD.

Intercellular interactions are mediated by surface receptors that often rely on Ca^2+^ for binding.^38^ Thus, we next tested whether extracellular Ca^2+^ plays a role in intracellular material acquisition by DCs. We supplemented the DC/KC or DC/B16 co-cultures with 5 mM EDTA to chelate extracellular Ca^2+^. We observed a significant inhibition of material transfer for both DC/KC (Figures 4A and S4A) and DC/B16 (Figure S4A) co-cultures. The inhibition reached maximum with 5 mM EDTA (Figure S4B). To determine whether intracellular Ca^2+^ also plays a role in intracellular monitoring, we supplemented the EDTA treated DC/B16 co-cultures or the Ca^2+^-free media with thapsigargin or BAPTA-AM. Adding thapsigargin, a non-competitive irreversible inhibitor of the endoplasmic reticular Ca^2+^ ATPase that is often used to deplete intracellular Ca^2+^,^39^ or BAPTA-AM, a cell membrane permeable Ca^2+^ chelator, to Ca^2+^-free media had additive effects, leading to an overall 60–70% inhibition of RNA transfer (Figure 4B). Thus, these data suggest a mechanism partially dependent on extracellular and intracellular Ca^2+^. Cadherins and integrins play an essential role in cell adhesion, synapse formation, and intercellular interactions in general, and some are Ca^2+^ dependent.^38^ Therefore, we next tested the contribution of certain, well-characterized cadherins and integrins to the RNA transfer. The DC/target cell co-cultures were supplemented with blocking antibodies to E-cadherin, CD11b, CD11c, RGD peptides (to block RGD-binding integrins), or ADH-1 (small molecule inhibitor of N-cadherin). We found no significant inhibition with any of the reagents tested (Figure S4C). The binding and potency of the antibodies and RGD peptides were confirmed prior to use (Figures S4D–S4F). These data suggest that the integrins and cadherins tested here do not play a substantial role in material transfer.

Protease mixtures, such as Pronase, that can digest a wide range of proteins, are often used to confirm the involvement of cell surface proteins in cellular interactions.^40^ To test whether RNA acquisition by DCs is Pronase sensitive, we treated the MutuDC1s with Pronase as previously described.^40^ The effect of Pronase digestion on cell surface proteins was confirmed by flow cytometry using markers such as CD8 (sensitive), CD11c, CD11b (partially sensitive), and MHC-II (resistant) (Figure S4D). Pronase treatment of the DCs caused slight (roughly 30%) but significant inhibition of RNA transfer (Figure 4C), supporting the involvement of a Pronase sensitive DC surface protein in material transfer. Pronase treatment of DCs has been reported to inhibit trogocytosis of target cell membrane by degrading class A scavenger receptor CD204, which can also be blocked by the molecule polyguanylic acid (PolyG).^40^^,^^41^ Adding PolyG to intact DC/B16 co-cultures led to roughly 50% inhibition of RNA transfer (Figure 4C). The difference in percent inhibition between Pronase and PolyG indicates that the two treatments act through different receptors, and that PolyG likely acts through a receptor other than CD204, as this receptor is Pronase sensitive^40^ and not expressed by the MutuDC1 cell line used here (Figure S4G). Blocking other scavenger receptors that are expressed by MutuDC1s, such as DEC205 and CD36L1 (SR-B1),^27^ resulted in negligible inhibition (Figure S4H). Pronase treatment in combination with PolyG caused near complete inhibition of RNA transfer (Figure 4D), supporting that these two treatments act through different receptors with partially redundant functions. Considering this redundancy, it is possible that the cadherins, integrins, and other receptors tested above do mediate material transfer, but that this effect can only be observed when they are blocked in combination with PolyG. After testing, apart from CD11c, we found that blocking candidate receptors in conjunction with PolyG had no effect over PolyG alone (Figures S4I–S4J). PolyG in combination with antibody binding MHC-II, an abundant surface protein that is not degraded by Pronase (Figure S4D), resulted in modest additive inhibition similar to anti-CD11c, indicating this inhibition likely reflects general steric effects and not a specific mechanism (Figure S4K).

Next, we probed whether Ca^2+^ works in concert with the Pronase-sensitive or PolyG-sensitive receptor. Interestingly, in combination with PolyG, but not with Pronase-treated DCs, EDTA almost completely inhibited RNA transfer (Figure 4D). PolyG+EDTA inhibition of RNA acquisition was effective with either KCs or B16s as donors (Figure S4L), and also inhibited the majority of protein transfer from B16s (Figure S4M). PolyG+EDTA also inhibited RNA transfer to human DCs from PBMCs (Figure S4N). Combining LY294, a PI3K inhibitor, with PolyG, but not EDTA showed additive effect in mouse cell cultures (Figures 4E and S4O). Thus, these data suggest a mechanism dependent on Ca^2+^ and that the Pronase-sensitive receptor on DCs is likely Ca^2+^- and PI3K-dependent. Overall, these data support that at least two sets of receptors mediate the RNA and protein acquisition by DCs.

### DCs present the antigen acquired through intracellular monitoring on both MHC-I and MHC-II

**Figure 5:**
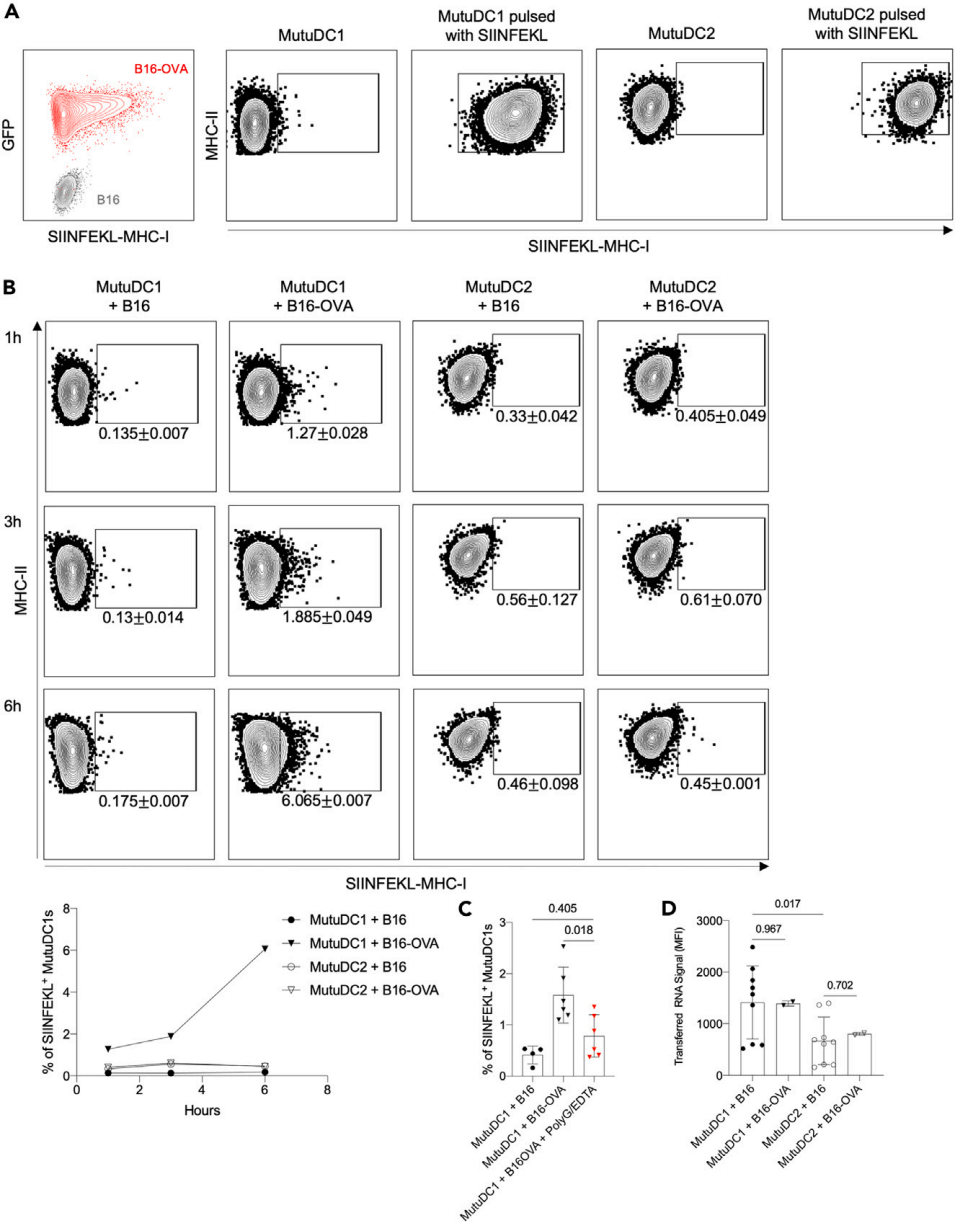
MutuDC1, but not MutuDC2 can cross-present the acquired ovalbumin (A) Control staining with SIINFEKL-MHC-I-specific antibody of B16, B16-OVA, MutuDC1, MutuDC1 pulsed with SIINFEKL, MutuDC2 and MutuDC2 pulsed with SIINFEKL. (B) MutuDC1 and MutuDC2 were co-cultured for the indicated time with B16 or B16-OVA and then the SIINFEKL-MHC-I levels determined by flow cytometry. Representative flow plots and summary graph (left lower corner) from one out of two experiments are shown with 2–3 technical replicates. (C) As in (B), but some of the MutuDC1 co-cultured with B16 or B16-OVA for 3 h were supplemented with PolyG/EDTA. Data from two independent experiments with 3 technical replicates were pooled. (D) MutuDC1 and MutuDC2 were co-cultured with B16 or B16-OVA labeled with SYTO62 for 45 min and then the transferred RNA signals (SYTO62) determined by flow cytometry. Data pooled from 3 independent experiments for B16, and one experiment for B16-OVA, with 2–3 technical replicates. Data are represented as mean ± SD.

We found that PolyG in concert with EDTA blocked monitoring with high efficiency, and that previously described antigen acquisition routes did not substantially contribute to RNA and protein transfer from target cells to DCs in our model. PolyG in combination with EDTA did not inhibit macropinocytosis (Figure S5A) but did inhibit phagocytosis in peritoneal macrophages (Figure S5B). However, because MutuDCs showed very poor phagocytic capability (Figure S5B), and inhibition of phagocytosis by cytochalasin D did not prevent RNA transfer (Figures 3E and S3H), PolyG/EDTA can be considered a specific inhibitor of intracellular monitoring in this system and used to address the immunological role of this unique antigen acquisition pathway. We first determined whether the acquired protein is presented on MHC-I. For this purpose, we co-cultured MutuDC1s, known to efficiently cross-present,^27^ or MutuDC2s, unable to cross-present,^42^ with B16 or B16-OVA cells for different time points. Then, we determined the presentation of the SIINFEKL peptide by DCs using peptide/MHC-I-specific antibody (Figure 5A). We found detectable levels of SIINFEKL peptides on the MutuDC1s co-cultured with B16-OVA, but not B16, as early as 1 h after co-incubation, which increased with time (Figure 5B). In contrast, with MutuDC2s we failed to detect any significant SIINFEKL presentation at any of the time points tested (Figure 5B). Inclusion of PolyG/EDTA in co-cultures significantly blocked SIINFEKL presentation by MutuDC1s (Figure 5C). The lack of cross-presentation was not due to the failure of MutuDC2 to perform intracellular monitoring; MutuDC2s, albeit less efficient than MutuDC1s, acquired significant amounts of RNA from both B16 and B16-OVA cells (Figure 5D). Thus, these data support that specific DC subsets specialized in cross-presentation can process and present antigen acquired through intracellular monitoring on MHC-I.

**Figure 6:**
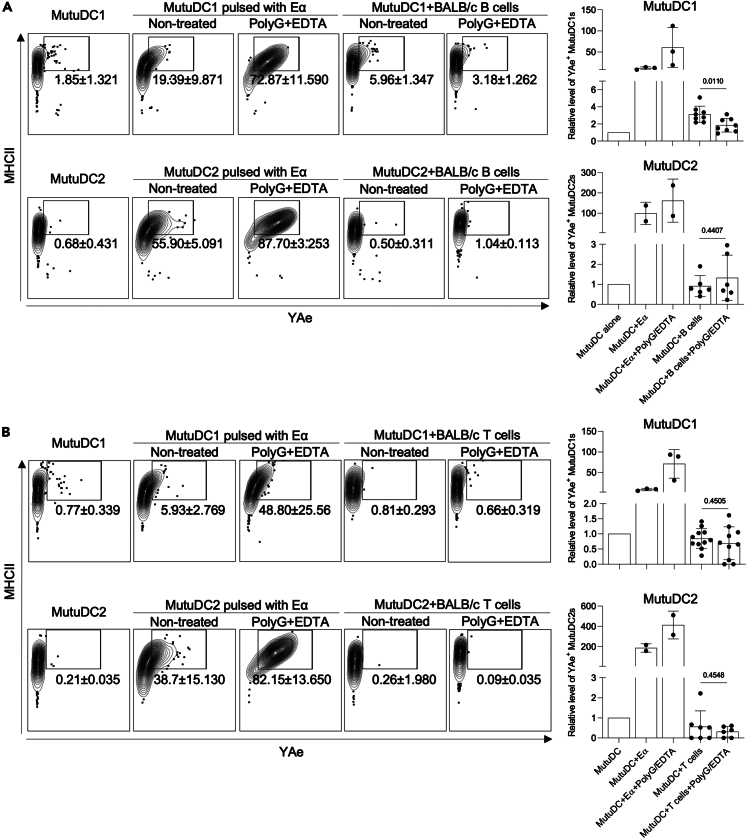
Materials acquired are presented on MHC-II (A) Top row, representative flow plots for YAe staining of MutuDC1 unmanipulated, pulsed with Eα peptide, or co-cultured for 3 h with BALB/c B cells either with or without PolyG/EDTA treatment. Second row, YAe staining of MutuDC2 unmanipulated, pulsed with Eα peptide, or co-cultured for 3 h with BALB/c B cells either with or without PolyG/EDTA treatment. Right: summary graph for MutuDC1 (top) and MutuDC2 (bottom). (B) As in (A), but BALB/c T cell were used. Data from three independent experiments with 2–3 technical replicates were pooled. Relative levels to DCs are shown. Data are represented as mean ± SD.

To determine whether the acquired antigens can be presented on MHC-II, we took advantage of the YAe antibody. The YAe antibody recognizes the Eα peptide presented in the context of I-Ab expressed by B6 mice. Eα peptide is derived from BALB/c MHC-II. Thus, we flow-sorted T and B cells from BALB/c skin-draining lymph nodes and co-cultured them with B6-derived MutuDC1 and MutuDC2 in the presence or absence of PolyG/EDTA. The rationale behind this setting was that the cells from BALB/c mice would serve as a source of Eα peptide. If the B6 DCs can take up MHC-II from the BALB/c cells, process, and present the resulting Eα on their MHC-II, then they should turn YAe positive. We found that MutuDC1s could present detectable amounts of Eα when co-cultured with B cells, but not with T cells, and that this presentation was significantly reduced in the presence of PolyG/EDTA (Figures 6A and 6B). In contrast, MutuDC2s did not present detectable amounts of Eα when co-cultured with either B or T cells (Figures 6A and 6B).

### DC maturation induced by inflammatory signals do not affect intracellular monitoring

**Figure 7:**
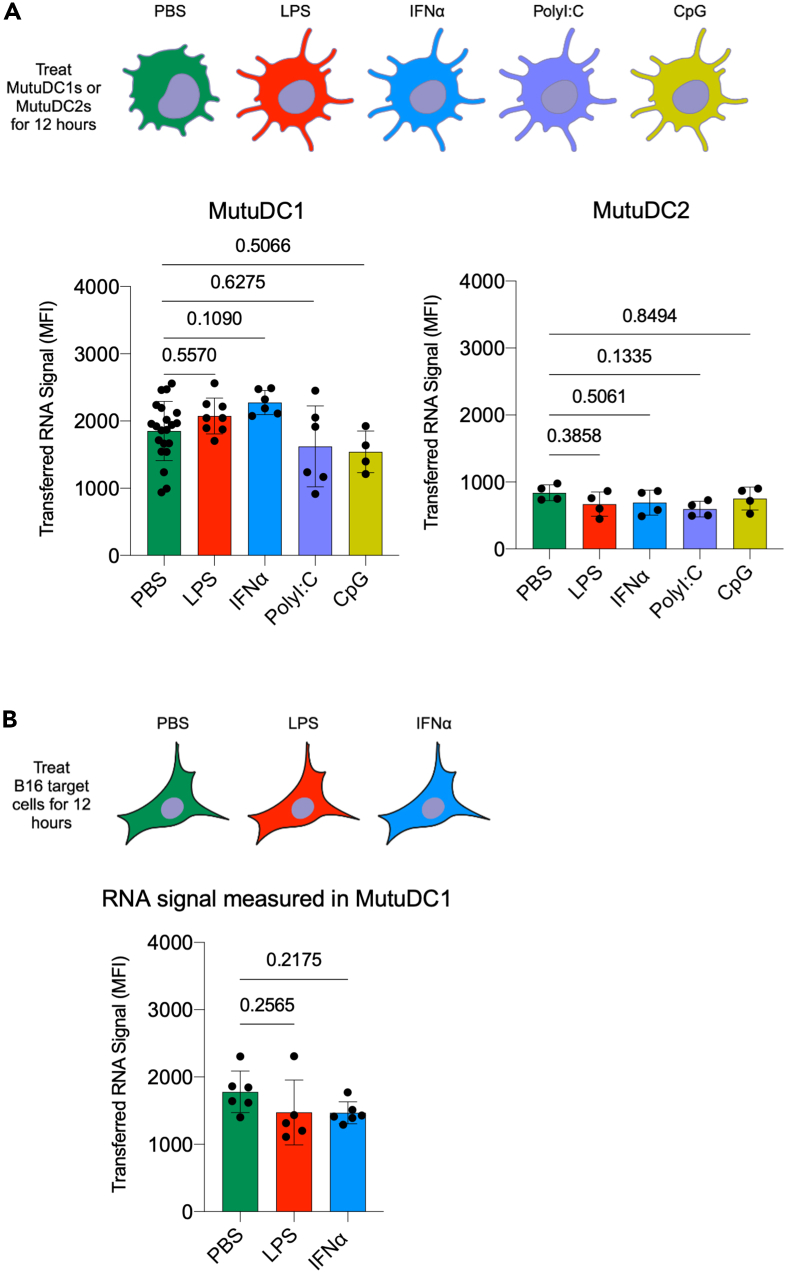
Inflammation does not alter RNA acquisition by DCs (A) MutuDC1s and MutuDC2s were exposed to 1 μg/mL LPS, 10 μg/mL IFNα, 5 μg/mL PolyI:C, or 0.5 μM CpG for 12 h and co-cultured with SYTO62-labeled B16 cells. The acquired RNA signal in DCs was determined by flow cytometry. (B) Like (A), but the B16 cells were treated as indicated. Data were pooled from two independent experiments, with 2–3 technical replicates. Data are represented as mean ± SD.

Maturation of DCs is thought to alter their capacity to acquire antigens through standard routes.^43^^,^^44^^,^^45^ To test whether acquisition of cytosolic material through intracellular monitoring is affected by maturation signals, we exposed the MutuDC1s and MutuDC2s for 12 h to 1 μg/mL LPS or 10 μg/mL IFNα or 5 μg/mL PolyI:C, or 0.5 μM CpG. Both cell lines express the receptors for these ligands, and they can respond to these stimuli by upregulating co-stimulatory markers.^27^^,^^42^ We also confirmed maturation through morphological changes and upregulation of specific markers. Representative data can be found in Figure S6. Then, the exposed and non-exposed DCs were compared side-by-side in an intracellular monitoring assay. We found no significant differences between treated and non-treated DCs in acquiring RNA from the target cells and between different treatments (Figure 7A). To mimic tissue inflammation and determine whether DCs entering an inflamed tissue could perform intracellular monitoring, we exposed the B16 target cells, known to express TLR-4,^46^ to LPS or IFNα for 12 h, then used them as target cells in our assay. The MutuDC1s were equally able to monitor both steady-state B16s and B16 exposed to inflammatory stimuli (Figure 7B). Thus, these data support that inflammatory conditions do not affect intracellular monitoring, which further separates it from other antigen acquisition routes.

## Discussion

Herein, we show that epidermal LCs overcome specific gene deletion when neighboring cells contain the missing gene product. Whereas MHC-II gene deletion in LCs results in a depletion of the corresponding protein, deleting genes coding for Cx43 and MyD88, expressed by neighboring KCs, does not decrease the quantity of gene products in knockout LCs. CXCR5 and MHC-II deletions are, however, overcome after LC migration to the CXCR5 and MHC-II rich skin draining lymph nodes.^24^ CD11b+CD103+ mLN DCs similarly overcome Cx43 conditional deletion, demonstrating that this trait is likely shared with DCs. After *in vitro* co-culture with RNA labeled donor cells, all primary DC subsets tested acquired RNA from neighboring cells to some extent. Co-cultures with different cell types revealed that DCs acquire RNA from a broad range of cell types, but cell types other than DCs acquire RNA at substantially lower rates. Human moDCs were also able to acquire RNA from autologous donor cells. Investigation into the mechanism of RNA transfer revealed it to be dependent on close contact and an active process, as physical separation, or direct contact while on ice results in near complete inhibition of material transfer. Live cell time-lapse confocal imaging shows DCs pressing dendrites into the membrane of donor cells and maintaining close contact. Actin cytoskeletal inhibition with cytochalasin D, which is known to prevent most forms of phagocytosis, endocytosis, trogocytosis, and the formation of tunneling nanotubes,^34^^,^^47^^,^^48^^,^^49^^,^^50^^,^^51^ does not substantially prevent transfer. RGD peptides or blocking antibodies against integrins commonly involved in endocytic process such as CD11c and CD11b^48^ also have no effect on transfer. EIPA, an inhibitor of macropinocytosis, and 1-heptanol, a gap junction inhibitor, also fail to prevent transfer. Instead, we find that transfer is partially inhibited by removing calcium from the media, PI3K inhibition, or by the introduction of PolyG into co-cultures. Combining PolyG with EDTA or Pronase treated DCs, but not EDTA with Pronase-treated DCs, is sufficient to block most of the transfer. Transferred material is successfully presented and cross-presented on MHC-II and MHC-I, and occurs between allogenic donor and acceptor cells. Inducing DC maturation with various inflammatory stimuli did not influence the material transfer observed here. Due to its discordance with conventional means of antigen uptake, we termed this route *intracellular monitoring* (ICM).

Our finding that LCs and DCs can overcome gene deficiencies has important implications for researchers utilizing conditional knockout models targeting DCs. Specifically, it emphasizes the importance of verifying protein depletion in addition to genetic recombination. Failing to do so may increase the likelihood of type II error, as the acquisition of protein from neighboring cells may lead researchers to incorrectly conclude that depleting the protein of interest has no effect, when in reality, lost protein was simply replaced through ICM. Furthermore, these data raise serious concerns regarding gene expression databases on DCs, which, based on our data, likely represent a mixture of mRNA from DCs and local cells. This highlights a need for the curation of RNA-seq data.

Aside from the immediate practical concerns surrounding ICM and conditional knockouts, ICM may also be relevant to important biological functions such as microenvironmental adaptation, immunosurveillance, and tolerance. Immune cell adaptation to the local microenvironment is a concept that has been extensively studied in macrophages, and refers to the dramatic shift in the chromatin landscape of macrophages in response to environmental queues such as retinoic acid or heme.^52^ These changes endow macrophages with functions necessary to operate properly in their local niche, and contribute to, instead of interrupt, the function of their resident organ.^53^ ICM may be providing a similar benefit to LCs. While we did not directly test the functionality of transferred protein in this study, one out of many viable explanations for LC’s possession of Cx43 is to prevent the disruption of wound healing, which is dependent on the direct transfer of Ca^2+^, IP_3_, and ATP through gap junctions and the ensuing calcium waves.^54^^,^^55^ These waves are projected to travel through LCs as well as KCs,^56^ supporting that LCs might acquire functional protein to help them adapt to their environment. Further studies investigating the fate and function of transferred material will help elucidate the roles of ICM in microenvironmental adaptation.

Considering DCs overcome the deficiency of multiple proteins, including ones they express on their own, it is possible that DCs continually and non-specifically conduct ICM. Among cell types tested, ICM was specific for DCs, and, to a lesser degree, macrophages. Its specificity for DCs and macrophages, combined with the finding that acquired protein is presented and cross-presented, points toward ICM being highly relevant to typical DC functions such as immunosurveillance and tolerance, and may explain the long-standing mystery of how DCs receive material from other cells for cross-presentation.^4^^,^^5^^,^^57^ DCs can monitor all donor cells tested, but more efficiently monitor CD45^−^cells and macrophages. If DCs use ICM to detect pathogens, monitoring macrophages with high efficiency would provide an evolutionary advantage, as these cells are often the first to encounter pathogens, and are more likely to contain a diverse pool of antigens. The low monitoring efficiency of the CD103+ cDC2 (CD11b+CD103+) mesenteric DCs, which migrate from the predominantly tolerogenic environment of the gut and are involved in T_reg_ and T_h17_ cell induction,^2^^,^^58^ supports this and argues against a role for ICM in maintaining tolerance, though this cannot be ruled out. On the other hand, it is also possible that while they are in the lamina propria of the gut, the very same DCs might possess high ICM capability, then downregulate it by the time they reach the mesenteric LNs to protect the cargo that requires tolerance induction. While this remains to be experimentally tested, we found that the opposite is true for LCs. LCs that have migrated to LNs are more efficient in ICM than their peripheral counterparts in the epidermis. Whether these site-specific differences have evolved to better serve tolerance induction or simply reflect that ICM is a tool for pathogen detection or that the monitored cell type and environment in the periphery will imprint a downstream program in the DCs, remains to be addressed.

From an evolutionary standpoint, it is logical that DCs would use ICM to detect pathogens. Intracellular pathogens have evolved complex and effective mechanisms to interfere with host cell processes and limit detection by the host immune system.^59^^,^^60^^,^^61^ Relying on material released or presented by infected cells is, therefore, not a dependable way to detect meddling pathogens. Direct presentation after infection also cannot be relied on, as not all viruses are DC tropic or highly cytopathic, and even if they are, DCs themselves could be subjected to pathogen immune evasion mechanisms, resulting in inefficient presentation. Thus, ICM may be a counter to the evading mechanisms developed by the pathogens. Our finding that ICM efficiency is unaffected by inflammatory stimuli or maturation is sensible in this context. A continuous and invariable monitoring system would be more difficult to distort than one that is regularly modulated. Further, if ICM facilitates inflammatory immune responses, our observation that DCs perform it in allogeneic and xenogeneic (unpublished observation) settings suggests it may play a role in organ rejection and be a valid therapeutic target.

While the exact mechanism of ICM remains to be determined, experiments conducted herein sufficiently differentiate from known processes of material transfer. Its contact-dependent nature rules out the uptake of extracellular material as a major contributing factor of RNA and protein transfer. Phagocytosis of dead or dying cells can be ruled out as dying cells are not prevented from detaching and coming in contact with DCs in our physical separation experiments. Cultured cells also maintained high viability throughout experimentation, and no donor cells or debris were observed in contact with DCs in physical separation experiments. TNTs are notoriously fragile and can be eliminated with doses as low as 50 nM Cytochalasin D,^62^ ruling out their involvement in material transfer. While some aspects of intracellular monitoring are reminiscent of trogocytosis, our findings are not consistent with this mechanism. Trogocytosis has been successfully inhibited by PolyG in DCs^40^ and LY294002 in other cell types,^47^ similar to what we observed, however, Harshyne et al. show that PolyG inhibits trogocytosis through blockade of Scavenger receptor A (CD204),^40^ which the MutuDC1 cells used in this study do not express. Further, the actin cytoskeleton is required for trogocytosis, whereas cytochalasin D fails to substantially prevent intracellular monitoring. Finally, trogocytosis almost exclusively refers to the transfer of membrane between cells,^63^ not the transfer of cytosolic material, further differentiating our observation of RNA and protein transfer from trogocytosis. Considering their size limitations, it is very unlikely that gap junctions would enable substantial RNA and protein transfer. Interestingly, in their study of oral tolerance, Mazzini et al., find that gap junctions only partially mediate transfer from macrophages to DCs, and note that “still-unknown mechanisms” may be contributing.^9^ In retrospect, it is likely that at least some of the material transfer from macrophages to DCs reported by Mazzini et al., was through ICM. ICM is further separated from other routes of antigen acquisition, such as phagocytosis, endocytosis, and macropinocytosis in that it is not significantly altered by the inflammatory signals that induce DCs maturation (Figure 7). These findings seem to contradict a widely accepted paradigm that during the maturation process the DCs downregulate antigen acquisition and upregulate antigen presentation.^43^^,^^44^^,^^45^ However, our data aligns with some of the *in vivo* findings showing that matured DCs remain efficient in acquiring soluble antigens.^64^ The fact that in our platform, we rarely detected phagocytic DCs (Figure S5B), and that ICM was minimally affected by actin cytoskeletal drug, further support that ICM might be the dominant route of acquisition of soluble cytosolic antigens both *in vitro* and *in vivo*.

We previously reported that human LCs, like their mouse counterpart, also contain detectable levels of Krt14 mRNA.^11^ Here, we further showed that human moDCs differentiated from CD14^+^ monocytes efficiently acquire RNA from PBMCs, and that RNA transfer can be significantly inhibited by PolyG/EDTA. These data support the translatability of our mouse data and indicate ICM may be a conserved process.

In summary, we show that a widely used research tool—Cre/Lox conditional gene knockout—may be inherently flawed when applied to DCs due to their ability to acquire material from neighboring cells through intracellular monitoring.

### Limitations of the study

Co-culture assays measuring the ability of various cell types to act as RNA donors or acceptors (Figure 2) would benefit from an expanded panel of cell types. Investigating alternative donor cells to the COCA KCs used here would be particularly helpful to ensure the trends identified in this manuscript are conserved in other settings.

Some functional ICM assays were only performed on cell lines. Further experiments will be needed to establish whether ICM has different functional characteristics in primary mouse and human DC subsets.

While we found that the combination of PolyG + EDTA blocks ICM with very high efficiency, it is not specific. Therefore, it cannot be used to discriminate between multiple routes of antigen acquisition in other cell types without first confirming that inhibitors of other antigen acquisition routes (cytochalasin D, heptanol, etc.) have poor efficacy.

We do not confirm whether transferred protein, or transferred RNA translated into protein is processed and presented, however, because of the short duration of the antigen presentation on MHC-I and MHC-II assays, it is likely the protein acquired through ICM that is being processed and presented by the DCs. It is expected that protein translation and presentation on MHC from transferred mRNA would take more time and, thus, contribute minimally to these assays. Nevertheless, the input from the two potential routes should be addressed experimentally.

A limitation of this manuscript is that Cytochalasin D is not a perfect inhibitor of endocytosis, which means that it does not rule out Massive Endocytosis, which is known not to require actin,^65^ or flotillin-dependent endocytosis, for which actin dependency is unknown,^66^ as possible mechanisms of transfer. Clathrin-mediated endocytosis is also sometimes independent of actin in mammals.^67^ However, considering that Massive Endocytosis has only been induced using whole-cell patch clamping and 150 μM calcium pulses, flotillin-dependent endocytosis is poorly defined, and clathrin-mediated endocytosis should be blocked by the concentrations of LY294002 used in this study,^68^^,^^69^ we maintain that the mechanism studied in this manuscript differs meaningfully from any previously described mode of material uptake/transfer.

Identifying the specific receptors mediating ICM will be critical to generate *in vivo* models to test the role of ICM in different aspects of immunity, such as immunosurveillance, tolerance induction, and organ transplant.

## STAR★Methods

### Key resources table

**Table:** 

REAGENT or RESOURCE	SOURCE	IDENTIFIER
**Antibodies**		

B220-PE	BioLegend	Cat# 103208; RRID:AB_312993
CD11b-PB	BioLegend	Cat#101224; RRID:AB_755988
CD11c-PB	BioLegend	Cat# 117322; RRID:AB_755986
CD14-PB (anti human)	BioLegend	Cat# 367121; RRID:AB_2687384
CD16/32	BioLegend	Cat# 101302; RRID:AB_312801
CD40-PE	BioLegend	Cat# 124610; RRID:AB_1134075
CD45.2-PE	BioLegend	Cat #109808; RRID:AB_313445
CD80-APC	BioLegend	Cat# 104713 RRID:AB_313134
CD86-BV605	BioLegend	Cat# 105037 RRID:AB_11204429
CD86-BV605 (anti-human)	BioLegend	Cat# 305430; RRID:AB_2563824
CD90.2-PE	BioLegend	Cat# 105308; RRID:AB_313179
CD103-BV650	BD	Cat# 748256; RRID:AB_2872685
CD204-PE	Fisher Scientific	Cat# 12204680; RRID:AB_2637409
CD205-Biotin	BioLegend	Cat# 138211; RRID:AB_10896431
CD207-APC	BioLegend	Cat# 144206; RRID:AB_2561998
F4/80-AF647	BioLegend	Cat# 123122
Fixable Viability Dye-eFluor780	Fisher Scientific	Cat# 5016966
H-2K^b^-BV421	BioLegend	Cat# 116525 RRID:AB_2876430
H-2K^b^ bound to SIINFEKL-PE	BioLegend	Cat# 141603; RRID:AB_10897938
HLA-DR-PerCP-Cy5.5 (anti-human)	BioLegend	Cat# 361710; RRID:AB_2750312
I-A/I-E-AF488	BioLegend	Cat #107616; RRID:AB_493523
Streptavidin-BV421	BioLegend	Cat# 405226
YAe-Biotin	Fisher Scientific	Cat# 5011908

**Biological samples**		

Skin, lymph nodes, spleen, peritoneal lavage	mouse	N/A

**Chemicals, peptides, and recombinant proteins**		

1-Heptanol	Fisher Scientific	Cat# AAA12793AE
2-Mercaptoethanol	Fisher Scientific	Cat# 21985023
2 μm Fluoresbrite® YG Carboxylate microspheres	PolySciences	Cat# 21636-1
5-(N-Ethyl-N-isopropyl)amiloride (EIPA)	Sigma Aldrich	Cat# A3085
8-chamber collagen I coated microscope slide	Corning	Cat# 354630
ADH1	MedChem Express	Cat# HY-13541
BAPTA-AM	Fisher Scientific	Cat# 502010390
BLT-1	MedChem Express	Cat# HY-116767
Calcein-AM dye	MedChem Express	Cat# HY-D0041
CellTrace CFSE	Fisher Scientific	Cat# 50591407
Chelex resin	Bio-Rad	Cat# 1421253
CnT-07 media	Fisher Scientific	Cat# NC9474150
CpG	InvivoGen	Cat# tlrl-2395f
Cytochalasin D	Millipore Sigma	Cat# 250255
DMSO	Sigma Aldrich	Cat# D2438-5X10ML
DreamTaq™ Hot Start Green PCR Master Mix	Fisher Scientific	FERK9022
EDTA	Fisher Scientific	Cat# AM9260G
Fetal bovine serum (FBS)	Fisher Scientific	Cat# MT35010CV, Lot: 14020001
HBSS	Fisher Scientific	Cat# 14170120
Human GM-CSF	PeproTech	Cat# 315-03
Human IL-4	PeproTech	Cat# 200-04
IFNa4	Made in house	Bouteau et al.,^70^
Iscove’s Modified Dulbecco’s Medium (IMDM)	Fisher Scientific	Cat# 31980097
Iscript Reverse Transcription supermix	Bio-Rad	Cat# 1708841
Itaq Universal SYBR green	Bio-Rad	Cat#1725121
LPS-EB Ultrapure	InvivoGen	Cat# tlrl-3pelps
LY294002	Tocris	Cat# 1130
MEM NEAA	Fisher Scientific	Cat# 11140050
Minimun Essential Medium (EMEM)	Caisson Labs	Cat# MEL19-500ML
NEAA	Fisher Scientific	Cat# BW06-174G
Newborn calf serum (NBCS)	Fisher Scientific	Cat# 26010074
Oligomycin A	Selleck Chemicals	Cat# S1478
Paraformaldehyde	Fisher Scientific	Cat# 50980495
Penicillin Streptomycin (Pen-strep)	Fisher Scientific	Cat# 15140122
Phosphate Buffered Saline (PBS)	Cytiva	Cat# SH30256.FS
Polyguanylic acid	Sigma Aldrich	Cat# P4404
PolyI:C	InvivoGen	Cat# tlrl-pic
Pronase protease	EMD Millipore	Cat# 53702-10KU
RGD peptide	Selleck Chemicals	Cat# S8008
RPMI	Cytiva	Cat# SH30096.FS
Sodium pyruvate	Fisher Scientific	Cat# 11360070
SYTO62 Red Fluorescent Nucleic Acid Stain	Fisher Scientific	Cat# S11344
Tag-it Violet	BioLegend	Cat# 425101
Thapsigargin	Fisher Scientific	Cat# NC9006970
TRITC-Dextran	Fisher Scientific	Cat# D1868
Trypsin-EDTA (0.25%)	Fisher Scientific	Cat# 25200072

**Critical commercial assays**		

Agilent Absolutely Rna Nanoprep Kit	Neta Scientific	Cat# 400753
EasySep™ Human Monocyte Isolation Kit	STEMCELL	Cat# 19319
EasySep™ Mouse CD11c Positive Selection Kit	STEMCELL	Cat# 18758
EasySep™ PE Positive Selection Kit II	STEMCELL	Cat# 17666
Genelute™ Mammalian Genomic DNA Miniprep Kits	Sigma-Aldrich	Cat# G1N70-1KT

**Experimental models: Cell lines**		

B16	Dr. Michael Gerner	
B16-OVA	Dr. Michael Gerner	
COCA	ECACC	Cat# 10112001
MutuDC1	Dr. Hans Acha-Orbea	
MutuDC2	Dr. Hans Acha-Orbea	

**Experimental models: Organisms/strains**		

hLangCre-Gja1^f/f^ mice	hLangCre from Dr. Daniel Kaplan; Gja1^f/f^ The Jackson Laboratory.	Stock# 008039
hLangCre-MyD88^f/f^ mice	Dr. D Kaplan	
hLangCre-CXCR5^f/f^ mice	hLangCre from Dr. Daniel Kaplan; CXCR5^f/f^ from Dr. Neil A. Mabbott and Anneli Peters.	
hLangCre-YFP^f/f^ mice	Dr. Daniel Kaplan	
hLangCre-MHC-II^f/f^ mice	Dr. Daniel Kaplan	
Balb/C mice	The Jackson Laboratory	Stock# 000651
C57BL/6 mice	The Jackson Laboratory	Stock# 000664

**Oligonucleotides**		

*gja1* CTTTGACTCTGATTACAGAGCTTAA (forward) for genotyping	Integrated DNA Technologies	Ref. # 277620819
*gja1* GTCTCACTGTTACTTAACAGCTTGA (reverse) for genotyping	Integrated DNA Technologies	Ref. # 277620817
*myd88* GGGAATAATGGCAGTCCTCTCCCAG (forward) for genotyping	Integrated DNA Technologies	
*myd88* CAGTCTCATCTTCCCCTCTGCC (reverse) for genotyping	Integrated DNA Technologies	
*cxcr5* AGGAGGCCATTTCCTCAGTT (forward)	Integrated DNA Technologies	
*cxcr5* GGCTTAGGGATTGCAGTCAG (reverse), and TTCCTTAGAGCCTGGAAAAGG (recombination)	Integrated DNA Technologies	
*myd88* Primetime primers for qPCR	Integrated DNA Technologies	Mm.PT.58.33389595
*gapdh* CTTTGTCAAGCTCATTTCCTGG (forward) for qPCR	Integrated DNA Technologies	
*gapdh* TCTTGCTCAGTGTCCTTG (reverse) for qPCR	Integrated DNA Technologies	
*gja1* TTCCTTTGACTTCAGCCTCC (forward) for qPCR	Integrated DNA Technologies	
*gja1h* CTTTGTCAAGCTCATTTCCTGG (reverse) for qPCR	Integrated DNA Technologies	

**Software and algorithms**		

GraphPad Prism	GraphPad Software	RRID:SCR_002798
FlowJo	BD	flowjo.com
ImageJ	ImageJ	ImageJ.net

**Other**		

8 mm Round coverslip	Electron Microscopy Sciences	Cat# 72296-08
96-well cell culture plates	Genesee Scientific	Cat# 25221
Carboxylate-modified Microspheres	Invitrogen	Cat# F8887
EX-LINE 75cm^2^ Polystyrene Tissue Culture Treated Flasks	Bio Basic	Cat# SP81186
Fisherbrand™ Surface Treated Sterile Tissue Culture Flasks	Fisher Scientific	Cat# FB012935, FB012937
ibidi μ-Slide 8 well	Fisher Scientific	Cat# NC0704855
uxcell Silicone O-Rings, 8mm × 5mm × 1.5mm	Amazon	Cat# B082SWJ5Y6

### Resource availability

#### Lead contact

Further information and requests for resources and reagents should be directed to and will be fulfilled by the lead contact, Botond Z. Igyártó (botond.igyarto@jefferson.edu).

#### Materials availability

This study did not generate new unique reagents.

#### Data and code availability

• The published article contains all datasets generated or analyzed during this study.
• This paper does not report original code.
• Any additional information required to reanalyze the data reported in this work paper is available from the lead contact upon request.

### Experimental model and study participant details

#### Ethics statement

Institutional Care and Use Committee at Thomas Jefferson University approved all mouse protocols. Protocol number: 02315.

#### Mice

hLangCre-YFP^f/f^,^71^ hLangCre-MHC-II^f/f 20^ and hLangCre-MyD88^f/f 22^ mice were previously described. hLangCre-Gja1^f/f^ (Cx43) and hLangCre-CXCR5^f/f^ mice were generated in house by crossing the huLangCre mice with Gja1^f/f^ mice (JAX stock#008039)^21^ and CXCR5^f/f 23^, respectively. All experiments were performed with 8–12 week old female and male mice. Mice were housed in microisolator cages and fed autoclaved food.

#### Cell lines

The COCA cell line was received from Sigma. The accompanying certificate of analysis certified it had been tested for mycoplasma using validated PCR primers (SOP ECC73), and Hoechst detection system (SOP ECC137). MutuDC1 and MutuDC2 cell lines were acquired directly from the lab of origin. No mycoplasma testing was done after receiving the cells in our facilities.

### Methods details

#### Experimental design

This study aimed to determine how DCs acquire cytosolic material from surrounding cells and define roles for this process. We designed and performed experiments using cellular immunology techniques, flow cytometry, qPCR, immunofluorescence microscopy, murine *in vivo* and *in vitro* models, and human models. The sample size and number of independent experiments are indicated in each figure legend.

#### Confirmation of genetic recombination

Epidermal cell suspensions were generated from hLangCre-Gja1^f/f^, hLangCre-MyD88^f/f^, and hLangCre-CXCR5^f/f^ mice^72^ and stained with eBioscience’s Fixable Viability Dye eFluor780, CD207 (4C7), I-A/I-E (M5/114.15.2), CD45.2 (104). LCs were sorted as live CD207+MHC-II + CD45.2+ cells, and KCs as triple negative. DNA was extracted with Genelute Mammalian Genomic DNA Miniprep kit according to the manufacturer’s protocol. The genetic recombination was verified using primers CTTTGACTCTGATTACAGAGCTTAA (forward) and GTCTCACTGTTACTTAACAGCTTGA (reverse) for *gja1*, which amplify a 600 bp segment in non-recombined Cre-mice, and no band in recombined mice. *myd88* genetic recombination was verified using primers GGGAATAATGGCAGTCCTCTCCCAG (forward) and CAGTCTCATCTTCCCCTCTGCC (reverse) for *myd88*, which amplify a 400 base pair segment in recombined cells. *cxcr5* genetic recombination was verified using primers AGGAGGCCATTTCCTCAGTT (forward), GGCTTAGGGATTGCAGTCAG (reverse), and TTCCTTAGAGCCTGGAAAAGG (recombination), which amplify a 292 base pair segment in recombined cells or a 375 base pair product in non-recombined cells.

#### MutuDC1/COCA keratinocyte coverslip co-culture experiments

COCA keratinocytes were allowed to adhere to 8 mm coverslips (Electron Microscopy Sciences) overnight in seeding media. Seeding media was then removed and cells were washed twice with PBS before media was replaced with CnT-07 and incubated overnight again until cells were nearly confluent. The following day, CnT-07 media was replaced with HBSS containing 50 nM SYTO62 RNA dye (Fisher Scientific), and cells were incubated for 20 min at 37°C before being washed twice with HBSS. Coverslips with stained keratinocytes were carefully moved using forceps to the bottom of a 48 well plate with cells facing up, and 25,000 MutuDC1 cells were added directly on top. Alternatively, an autoclaved silicone O-ring (width 1.5 mm) was placed in the well, and 25,000 MutuDC1s were added inside of it and allowed to settle. Keratinocyte coated coverslips were then placed on top of the O-ring with cells facing down. Wells were filled with DC media so that suspended coverslips were completely submerged. After a 45 min incubation at either 37°C or on ice, MutuDC1 cells were resuspended by pipetting up and down, and transferred RNA was measured by flow cytometry. The same protocol was conducted using ibidi 8 chamber slides (Fisher Scientific) to allow for confocal imaging with the exception that a positive control condition was included (Dye in Media) where SYTO62 was not washed out. Images were taken on a Nikon A1R Confocal microscope using a Plan Fluor 40× Oil objective at the end of the 45 min incubation. The amount of transferred RNA was measured in ImageJ by calculating the mean far-red pixel intensity (SYTO62 signal) contained within regions of high green channel signal (representative GFP^+^ MutuDC1s). Briefly, green channel images were converted to 8-bit and thresholded appropriately. Watersheding was used to parse clumped cells, then the analyze particle’s function was used to identify Regions of Interests corresponding to area within MutuDC1 cells. These ROIs were then applied to the far-red channel and mean pixel intensity was calculated.

#### Study of RNA transfer in the presence of inhibitors

Once nearing confluence, MutuDC1, B16s, or COCA cells were harvested as described above and suspended in 1 mL of their respective media. Donor cells – either COCA cells or B16s -- were pelleted and resuspended at 10^6^ cells/ml in pre-warmed HBSS containing 200 nM SYTO62 dye, then incubated at 37°C for 20 min. Donor cells were then washed twice with ice-cold PBS and resuspended in DC media with inhibitor or vehicle. At this time, MutuDC1 cells were also resuspended in media containing inhibitor or vehicle. Cells were protected from light, then left on ice for 30 min in the presence of inhibitor. Reagents used in this study include: 8 μM Cytochalasin D (Millipore Sigma), 10 μM Oligomycin A (Selleck Chemicals), 32 μM 5-(N-Ethyl-N-isopropyl)amiloride (EIPA) (Sigma Aldrich), 5 mM 1-Heptanol (Fisher Scientific), 5 mM EDTA (Fisher Scientific), 2 μM Thapsigargin (Fisher Scientific), 50 μM BAPTA-AM (Fisher Scientific), 50 μM LY294002 (Tocris), 350 nM ADH-1 (MedChem Express), 500 μg/mL Polyguanylic acid (Sigma-Aldrich), 1 mg/mL RGD peptide (Selleck Chemicals), and BLT-1 (MedChemExpress). Calcium was removed from DC media using Chelex resin (Bio-Rad). Blocking antibodies specific for the following proteins were used: 2.5 μg/mL CD11b (M1/70, BioLegend), 2.5 μg/mL CD11c (N418, BioLegend), 1 μg/mL CD204 (M204PA, Fisher Scientific), and 2.5 μg/mL CD205 (NLDC-145, BioLegend). In some cases, MutuDC1 cells were treated with 32 μg/mL Pronase (EMD Millipore) for 20 min at 37°C.^40^ Both COCA and MutuDC1 cells were then counted and combined into a 96-well plate containing media with inhibitor or appropriate vehicle. Eighty thousand COCA keratinocytes or B16 cells were combined with 10,000 MutuDC1s. In some experiments, primary cells were used for co-culture after being sorted as described. In these experiments, 10,000 acceptor cells were stained with 5 μM CFSE (Fisher Scientific) for 5 min on ice and mixed with 80,000 donor cells. Once plated, cells were mixed and moved directly from ice to a 37°C, 5% CO_2_ incubator, and incubated for 45 min. After incubation, cells were moved back to ice, resuspended by pipetting, filtered through a 50 μm filter, and run on flow cytometer.

#### Live cell imaging using confocal microscopy

Cultures were imaged in ibidi 8 chamber slides held in a humidified chamber at 37°C and 5% CO_2_.

#### Cross-presentation experiment

B16 or B16-OVA cells were co-cultured with MutuDC1s or MutuDC2s as triplicates in a U-bottom 96-well plate in a CO_2_ incubator for 1, 3 and 6 h. Some of the 3 h cultures were supplemented with a standard dose of PolyG/EDTA. After incubation the cells were washed, stained with eBioscience’s Fixable Viability Dye eFluor780, I-A/I-E (M5/114.15.2) and SIINFEKL/MHC-I (eBio25-D1.16) and analyzed by flow cytometer.

#### Presentation on MHC-II

BALB/c T and B cells purified by flow cytometry were co-cultured with MutuDC1s or MutuDC2s for 3 h in the absence or presence of standard dose of PolyG/EDTA. After incubation the cells were washed, stained with eBioscience’s Fixable Viability Dye eFluor780, I-A/I-E (M5/114.15.2) and YAe (eBioY-Ae) antibodies and analyzed by flow cytometer.

#### Study of RNA transfer in inflammatory conditions

MutuDC1s and MutuDC2s were exposed to 1 μg/mL LPS, 10 μg/mL IFNα, 5 μg/mL PolyI:C, or 0.5 μM CpG for 12 h, then rinsed and co-cultured with SYTO62-labeled B16 cells for 45 min. The SYTO62 signal in control and treated MutuDC1 and MutuDC2 was determined by flow cytometry. The activation of the DCs was confirmed based on morphological changes by microscopy and by flow cytometry with the following markers: CD40, CD80, CD86, and H2-K^b^ (BioLegend). In separate experiments, the B16 cells were left untreated or exposed to 1 μg/mL LPS or 10 μg/mL IFNα for 12 h, then labeled with SYTO62 dye and co-cultured with unmanipulated MutuDC1s.

#### Cell sorting

T and B cells were sorted from the spleen of wild-type C57BL/6 mice. A single cells suspension was generated and stained with eBioscience’s Fixable Viability Dye eFluor780 and anti-CD90.2 (30-H12, BioLegend) and anti-CD19 (6D5, BioLegend). CD90.2+ cells were considered T cells and CD19^+^ cells were considered B cells. Cells were sorted on a BD FACSAria II sorter.

CD45^−^cells were sorted from the dermis of a wild-type C57BL/6 mouse. A single cell suspension was generated and stained with eBioscience’s Fixable Viability Dye eFluor780 and anti-CD45.2 (104, BioLegend).

Lymph node cell suspensions were generated from hLangCre-YFP^f/f^ mice and enriched for CD11c+ cells using EasySep Mouse CD11c Positive Selection Kit (StemCell, #18758). Post enrichment cells were stained with eBioscience’s Fixable Viability Dye eFluor780 and the following antibodies: CD11c (N418), I-A/I-E (M5/114.15.2), CD103 (2E7) and CD207 (4C7). Resident DCs were gated as live CD11c^hi^ MHCII^med^, migratory LCs were gated as live MHCII^hi^CD11c^med^YFP+, cDC1 were gated as live MHCII^hi^CD11c^med^CD103+CD207+, cDC2s were gated as live MHCII^hi^CD11c^med^CD207-, mesenteric DCs were gated as live YFP+. Cells were sorted on a BD FACSAria II sorter.

Epidermal cell suspensions were generated from hLangCre-YFP^f/f^ mice. Cell suspensions were stained with anti-CD45.2 (104) PE antibody and enriched for LCs using EasySep PE Positive Selection Kit II (StemCell). Post enrichment cells were stained with eBioscience’s Fixable Viability Dye eFluor780 and sorted for live YFP+ cells on a BD FACSAria II sorter.

Cells from a peritoneal lavage were stained with viability eBioscience’s Fixable Viability Dye eFluor780, F4/80 (BM8), and CD11b (M1/70). Macrophages were gated as F4/80^hi^CD11b^hi^ cells and sorted on a BD FACSAria II sorter.

#### Quantitative PCR

Cells were sorted directly into 100 μL of lysis buffer supplemented with beta-mercaptoethanol from the Agilent Absolutely Rna Nanoprep Kit (Neta Scientific) and RNA was extracted according to the manufacturer’s protocol. Iscript Reverse Transcription supermix (Bio-Rad) was used to generate cDNA. qPCR reactions were conducted using 1 μL of cDNA product in 10 μL total reaction volume using Itaq Universal SYBR green (Bio-Rad) with primers at a concentration of 500 nM. Primers used are as follows: IDT *myd88* Primetime primers (IDT, Mm.PT.58.33389595), *gapdh* Reverse: TCTTGCTCAGTGTCCTTG, *gapdh* Forward: CTTTGTCAAGCTCATTTCCTGG, *gja1* reverse: CGTGGAGTAGGCTTGGAC *gja1* forward: TTCCTTTGACTTCAGCCTCC.

#### Generation of human DCs and co-culture with autologous PBMCs

To make human myeloid-derived DCs, 2 × 10^6^ human CD14^+^CD16^−^blood monocytes isolated with EasySep Human Monocyte Isolation Kit (StemCell) were cultured in six-well plates (2 mL per well) in complete RPMI 1640 medium +10% FBS +10 ng/mL human IL-4 (PeproTech) + 100 ng/mL human GM-CSF (PeproTech). Half of the medium was changed at day 2 and at day 4, maintaining the same concentration of IL-4 and GM-CSF. Immature DCs were collected on day 5 and analyzed using a flow cytometer (CD14, HLA-DR and CD86). After confirmation the remaining DCs were used for intracellular monitoring. Briefly, the DCs were labeled with CFSE and co-cultured with autologous PBMCs labeled with RNA dye in the presence or absence of PolyG/EDTA for 45 min. The RNA acquisition by DCs from PBMCs was confirmed by flow cytometer.

#### Actin polymerization inhibition assay

100,000 MutuDC1 cells were plated in each chamber of a removable 8 chamber slide and left to incubate for 2 h at 37°C to allow attachment. Media was then aspirated off and replaced with media containing 4 μM Cytochalasin D or DMSO control and incubated for an additional 30 min. The media was then aspirated off, and cells were washed once with PBS and fixed with 4% paraformaldehyde (EM Grade, Fisher Scientific) in PBS for 20 min. After fixation, the paraformaldehyde was removed, and cells were washed two more times with PBS. 183 nM Phalloidin labeled with rhodamine working solution was then added, and cells were incubated for 60 min at room temperature. The cells were washed twice more with PBS. The removable chambers were detached, and coverslips placed over the cells using mounting media for imaging.

#### Phagocytosis assay

2 μm Carboxylate microspheres (Invitrogen F8887) were incubated overnight in 1% BSA at room temperature with gentle shaking. The following day, beads were washed twice with water and resuspended In DC media containing inhibitor or vehicle control at a concentration of 0.002% solids. Sorted murine peritoneal macrophages or MutuDC1 cells were then resuspended in the bead containing media and incubated for 1 h at 37°C or on ice. For cytochalasin D conditions, cells were incubated in cytochalasin D containing media for 30 min prior to incubation with beads. After incubation, cells were washed twice with ice-cold PBS, resuspended in staining media, and run by flow cytometry.

#### TRITC-dextran uptake

MutuDC1 cells were incubated in 32 μM EIPA or vehicle control for 30 min on ice. Cells were then centrifuged and resuspended in HBSS containing 16 μM or 32 μM EIPA or vehicle and 20 μg/mL 10 kDa TRITC-Dextran (Fisher Scientific) and incubated at 37°C for 30 min. After incubation, cells were washed twice with PBS, then resuspended in Trypsin-EDTA (0.25%) for 5 min to remove any Dextran adherent to the outside of the cell. Cells were then washed twice more and analyzed by flow cytometry.

#### Gap junction calcein transfer assay

COCA keratinocytes were separated into two aliquots. One aliquot was stained with 10 μM Tag-it Violet (BioLegend) diluted in HBSS for 5 min on ice at a cell concentration of 1 million cells per milliliter. After incubation, 5x volume of serum containing media was added to the tube and cells were incubated for an additional 5 min on ice. Cells were then centrifuged at 240 g for 7 min, washed once with PBS, and finally resuspended in media. The second aliquot of cells was stained with 100 nM Calcein-AM dye diluted in HBSS for 5 min on ice at a cell concentration of 1 million cells per milliliter. To potentiate intracellular esterases activity, cells were incubated for an additional 20 min at 37°C. Centrifuging at 240 g for 7 min, cells were washed twice with PBS and finally resuspended in media. To ensure cell contact, 75,000 of each Tag-it Violet and Calcein labeled cells were combined in the wells of a 96 well flat bottom plate in media containing either 5 mM 1-Heptanol or vehicle control and incubated for 2 h at 37°C. Calcein transfer to Tag-it Violet+ cells was measured by flow cytometry by comparing to unstained controls.

#### RGD peptide adherence assay

B16 cells were suspended in cold B16 media with or without 1 mg/mL RGD peptides. Two hundred and fifty thousand cells were then plated per chamber of an 8-chamber collagen I-coated microscope slide (Corning). Cells were incubated for 45 min at 37°C and 5% CO_2_. After incubation, the media was removed and replaced with PBS, and cells were imaged. The PBS was then removed and combined with initial media as the non-adherent fraction, and 200 μL warmed Trypsin-EDTA (0.25%) was added to each well and incubated for 5 min. Two hundred microliters of media were then added to each chamber and pipetted up and down to ensure the removal of all adherent cells. This was considered the adherent fraction.

### Quantification and statistical analysis

Data were analyzed by unpaired two-tailed Student’s *t* test for parametric data, and one-way analysis of variance (ANOVA) followed by Tukey’s post hoc test for multiple comparisons. Data normality was determined using the Shapiro-Wilks test. Data displayed as Mean ± SD. Additional information is reported in figure legends. GraphPad Prism software was used for the analyses (GraphPad Software, La Jolla, CA).

## Supplemental information

Document S1. Figures S1‒S6

## References

[1] Mellman I., Steinman R.M. (2001). Dendritic cells: specialized and regulated antigen processing machines. Cell.

[2] Yin X., Chen S., Eisenbarth S.C. (2021). Dendritic Cell Regulation of T Helper Cells. Annu. Rev. Immunol..

[3] Sallusto F., Cella M., Danieli C., Lanzavecchia A. (1995). Dendritic cells use macropinocytosis and the mannose receptor to concentrate macromolecules in the major histocompatibility complex class II compartment: downregulation by cytokines and bacterial products. J. Exp. Med..

[4] Marañón C., Desoutter J.F., Hoeffel G., Cohen W., Hanau D., Hosmalin A. (2004). Dendritic cells cross-present HIV antigens from live as well as apoptotic infected CD4+ T lymphocytes. Proc. Natl. Acad. Sci. USA.

[5] Ramirez M.C., Sigal L.J. (2002). Macrophages and Dendritic Cells Use the Cytosolic Pathway to Rapidly Cross-Present Antigen from Live, Vaccinia-Infected Cells. J. Immunol..

[6] Matheoud D., Perié L., Hoeffel G., Vimeux L., Parent I., Marañón C., Bourdoncle P., Renia L., Prevost-Blondel A., Lucas B. (2010). Cross-presentation by dendritic cells from live cells induces protective immune responses in vivo. Blood.

[7] Thomas M.P., Liu X., Whangbo J., McCrossan G., Sanborn K.B., Basar E., Walch M., Lieberman J. (2015). Apoptosis Triggers Specific, Rapid, and Global mRNA Decay with 3’ Uridylated Intermediates Degraded by DIS3L2. Cell Rep..

[8] van Dinther D., Veninga H., Iborra S., Borg E.G.F., Hoogterp L., Olesek K., Beijer M.R., Schetters S.T.T., Kalay H., Garcia-Vallejo J.J. (2018). Functional CD169 on Macrophages Mediates Interaction with Dendritic Cells for CD8+ T Cell Cross-Priming. Cell Rep..

[9] Mazzini E., Massimiliano L., Penna G., Rescigno M. (2014). Oral tolerance can be established via gap junction transfer of fed antigens from CX3CR1^+^ macrophages to CD103^+^ dendritic cells. Immunity.

[10] Wattrus S.J., Smith M.L., Rodrigues C.P., Hagedorn E.J., Kim J.W., Budnik B., Zon L.I. (2022). Quality assurance of hematopoietic stem cells by macrophages determines stem cell clonality. Science.

[11] Su Q., Igyártó B.Z. (2019). Keratinocytes Share Gene Expression Fingerprint with Epidermal Langerhans Cells via mRNA Transfer. J. Invest. Dermatol..

[12] Bettadapur A., Miller H.W., Ralston K.S. (2020). Biting off what can be chewed: Trogocytosis in health, infection, and disease. Infect. Immun..

[13] Wakim L.M., Bevan M.J. (2011). Cross-dressed dendritic cells drive memory CD8+ T-cell activation after viral infection. Nature.

[14] Kroger C.J., Spidale N.A., Wang B., Tisch R. (2017). Thymic Dendritic Cell Subsets Display Distinct Efficiencies and Mechanisms of Intercellular MHC Transfer. J. Immunol..

[15] Perry J.S.A., Russler-Germain E.V., Zhou Y.W., Purtha W., Cooper M.L., Choi J., Schroeder M.A., Salazar V., Egawa T., Lee B.-C. (2018). transfer of cell-surface antigens by scavenger receptor CD36 promotes thymic regulatory T cell receptor repertoire development and allo-tolerance. Immunity.

[16] Rustom A., Saffrich R., Markovic I., Walther P., Gerdes H.-H. (2004). Nanotubular highways for intercellular organelle transport. Science.

[17] Hase K., Kimura S., Takatsu H., Ohmae M., Kawano S., Kitamura H., Ito M., Watarai H., Hazelett C.C., Yeaman C., Ohno H. (2009). M-Sec promotes membrane nanotube formation by interacting with Ral and the exocyst complex. Nat. Cell Biol..

[18] Zaccard C.R., Watkins S.C., Kalinski P., Fecek R.J., Yates A.L., Salter R.D., Ayyavoo V., Rinaldo C.R., Mailliard R.B. (2015). CD40L induces functional tunneling nanotube networks exclusively in dendritic cells programmed by mediators of type 1 immunity. J. Immunol..

[19] Neijssen J., Herberts C., Drijfhout J.W., Reits E., Janssen L., Neefjes J. (2005). Cross-presentation by intercellular peptide transfer through gap junctions. Nature.

[20] Igyártó B.Z., Jenison M.C., Dudda J.C., Roers A., Müller W., Koni P.A., Campbell D.J., Shlomchik M.J., Kaplan D.H. (2009). Langerhans cells suppress contact hypersensitivity responses via cognate CD4 interaction and langerhans cell-derived IL-10. J. Immunol..

[21] Liao Y., Day K.H., Damon D.N., Duling B.R. (2001). Endothelial cell-specific knockout of connexin 43 causes hypotension and bradycardia in mice. Proc. Natl. Acad. Sci. USA.

[22] Haley K., Igyártó B.Z., Ortner D., Bobr A., Kashem S., Schenten D., Kaplan D.H. (2012). Langerhans cells require MyD88-dependent signals for Candida albicans response but not for contact hypersensitivity or migration. J. Immunol..

[23] Bradford B.M., Reizis B., Mabbott N.A. (2017). Oral prion disease pathogenesis is impeded in the specific absence of CXCR5-expressing dendritic cells. J. Virol..

[24] Yao C., Kaplan D.H. (2018). Langerhans cells transfer targeted antigen to dermal dendritic cells and acquire major histocompatibility complex II in vivo. J. Invest. Dermatol..

[25] Doebel T., Voisin B., Nagao K. (2017). Langerhans Cells - The Macrophage in Dendritic Cell Clothing. Trends Immunol..

[26] Welty N.E., Staley C., Ghilardi N., Sadowsky M.J., Igyártó B.Z., Kaplan D.H. (2013). Intestinal lamina propria dendritic cells maintain T cell homeostasis but do not affect commensalism. J. Exp. Med..

[27] Fuertes Marraco S.A., Grosjean F., Duval A., Rosa M., Lavanchy C., Ashok D., Haller S., Otten L.A., Steiner Q.-G., Descombes P. (2012). Novel Murine Dendritic Cell Lines: A Powerful Auxiliary Tool for Dendritic Cell Research. Front. Immunol..

[28] Segrelles C., Holguín A., Hernández P., Ariza J.M., Paramio J.M., Lorz C. (2011). Establishment of a murine epidermal cell line suitable for in vitro and in vivo skin modelling. BMC Dermatol..

[29] Ståhl A.L., Johansson K., Mossberg M., Kahn R., Karpman D. (2019). Exosomes and microvesicles in normal physiology, pathophysiology, and renal diseases. Pediatr. Nephrol..

[30] Haimovich G., Ecker C.M., Dunagin M.C., Eggan E., Raj A., Gerst J.E., Singer R.H. (2017). Intercellular mRNA trafficking via membrane nanotube-like extensions in mammalian cells. Proc. Natl. Acad. Sci. USA.

[31] Shchepina L.A., Pletjushkina O.Y., Avetisyan A.V., Bakeeva L.E., Fetisova E.K., Izyumov D.S., Saprunova V.B., Vyssokikh M.Y., Chernyak B.V., Skulachev V.P. (2002). Oligomycin, inhibitor of the F0 part of H+-ATP-synthase, suppresses the TNF-induced apoptosis. Oncogene.

[32] Zurzolo C. (2021). Tunneling nanotubes: Reshaping connectivity. Curr. Opin. Cell Biol..

[33] Kapetanovic R., Nahori M.-A., Balloy V., Fitting C., Philpott D.J., Cavaillon J.-M., Adib-Conquy M. (2007). Contribution of Phagocytosis and Intracellular Sensing for Cytokine Production by Staphylococcus aureus -Activated Macrophages. Infect. Immun..

[34] Magae J., Nagi T., Takaku K., Kataoka T., Koshino H., Uramoto M., Nagai K. (1994). Screening for Specific Inhibitors of Phagocytosis of Thioglycollate-elicited Macrophages. Biosci. Biotechnol. Biochem..

[35] Koivusalo M., Welch C., Hayashi H., Scott C.C., Kim M., Alexander T., Touret N., Hahn K.M., Grinstein S. (2010). Amiloride inhibits macropinocytosis by lowering submembranous pH and preventing Rac1 and Cdc42 signaling. J. Cell Biol..

[36] Lin X.P., Mintern J.D., Gleeson P.A. (2020). Macropinocytosis in Different Cell Types: Similarities and Differences. Membranes.

[37] Balla P., Maros M.E., Barna G., Antal I., Papp G., Sapi Z., Athanasou N.A., Benassi M.S., Picci P., Krenacs T. (2015). Prognostic impact of reduced connexin43 expression and gap junction coupling of neoplastic stromal cells in giant cell tumor of bone. PLoS One.

[38] Kim S.A., Tai C.Y., Mok L.P., Mosser E.A., Schuman E.M. (2011). Calcium-dependent dynamics of cadherin interactions at cell-cell junctions. Proc. Natl. Acad. Sci. USA.

[39] Inesi G., Sagara Y. (1992). Thapsigargin, a high affinity and global inhibitor of intracellular Ca2+ transport ATPases. Arch. Biochem. Biophys..

[40] Harshyne L.A., Zimmer M.I., Watkins S.C., Barratt-Boyes S.M. (2003). A Role for Class A Scavenger Receptor in Dendritic Cell Nibbling from Live Cells. J. Immunol..

[41] Harvey B.P., Raycroft M.T., Quan T.E., Rudenga B.J., Roman R.M., Craft J., Mamula M.J. (2014). Transfer of antigen from human B cells to dendritic cells. Mol. Immunol..

[42] Pigni M., Ashok D., Stevanin M., Acha-Orbea H. (2018). Establishment and Characterization of a Functionally Competent Type 2 Conventional Dendritic Cell Line. Front. Immunol..

[43] Garrett W.S., Chen L.M., Kroschewski R., Ebersold M., Turley S., Trombetta S., Galán J.E., Mellman I. (2000). Developmental control of endocytosis in dendritic cells by Cdc42. Cell.

[44] West M.A., Prescott A.R., Eskelinen E.L., Ridley A.J., Watts C. (2000). Rac is required for constitutive macropinocytosis by dendritic cells but does not control its downregulation. Curr. Biol..

[45] Ho Y.H.S., Cai D.T., Huang D., Wang C.C., Wong S.H. (2009). Caspases regulate VAMP-8 expression and phagocytosis in dendritic cells. Biochem. Biophys. Res. Commun..

[46] Andreani V., Gatti G., Simonella L., Rivero V., Maccioni M. (2007). Activation of Toll-like receptor 4 on tumor cells in vitro inhibits subsequent tumor growth in vivo. Cancer Res..

[47] Miyake K., Karasuyama H. (2021). The Role of Trogocytosis in the Modulation of Immune Cell Functions. Cells.

[48] Rubartelli A., Poggi A., Zocchi M.R. (1997). The selective engulfment of apoptotic bodies by dendritic cells is mediated by the αvβ3 integrin and requires intracellular and extracellular calcium. Eur. J. Immunol..

[49] Albert M.L., Pearce S.F., Francisco L.M., Sauter B., Roy P., Silverstein R.L., Bhardwaj N. (1998). Immature Dendritic Cells Phagocytose Apoptotic Cells via αvβ5 and CD36, and Cross-present Antigens to Cytotoxic T Lymphocytes. J. Exp. Med..

[50] Bukoreshtliev N.V., Wang X., Hodneland E., Gurke S., Barroso J.F.V., Gerdes H.-H. (2009). Selective block of tunneling nanotube (TNT) formation inhibits intercellular organelle transfer between PC12 cells. FEBS Lett..

[51] Biran A., Perelmutter M., Gal H., Burton D.G.A., Ovadya Y., Vadai E., Geiger T., Krizhanovsky V. (2015). Senescent cells communicate via intercellular protein transfer. Genes Dev..

[52] Lavin Y., Winter D., Blecher-Gonen R., David E., Keren-Shaul H., Merad M., Jung S., Amit I. (2014). Tissue-resident macrophage enhancer landscapes are shaped by the local microenvironment. Cell.

[53] Lazarov T., Juarez-Carreño S., Cox N., Geissmann F. (2023). Physiology and diseases of tissue-resident macrophages. Nature.

[54] Leybaert L., Sanderson M.J. (2012). Intercellular Ca(2+) waves: mechanisms and function. Physiol. Rev..

[55] Hudson L., Begg M., Wright B., Cheek T., Jahoda C.A.B., Reynolds N.J. (2021). Dominant effect of gap junction communication in wound-induced calcium-wave, NFAT activation and wound closure in keratinocytes. J. Cell. Physiol..

[56] Donati V., Peres C., Nardin C., Scavizzi F., Raspa M., Ciubotaru C.D., Bortolozzi M., Pedersen M.G., Mammano F. (2022). Calcium signaling in the photodamaged skin: In vivo experiments and mathematical modeling. Funct..

[57] Freigang S., Egger D., Bienz K., Hengartner H., Zinkernagel R.M. (2003). Endogenous neosynthesis vs. cross-presentation of viral antigens for cytotoxic T cell priming. Proc. Natl. Acad. Sci..

[58] Scott C.L., Aumeunier A.M., Mowat A.M. (2011). Intestinal CD103+ dendritic cells: master regulators of tolerance?. Trends Immunol..

[59] Reddick L.E., Alto N.M. (2014). Bacteria Fighting Back: How Pathogens Target and Subvert the Host Innate Immune System. Mol. Cell.

[60] Sharp T.M., Estes M.K. (2010). An inside job: subversion of the host secretory pathway by intestinal pathogens. Curr. Opin. Infect. Dis..

[61] Alcami A., Koszinowski U.H. (2000). Viral mechanisms of immune evasion. Immunol. Today.

[62] Bittins M., Wang X. (2017). TNT-Induced Phagocytosis: Tunneling Nanotubes Mediate the Transfer of Pro-Phagocytic Signals From Apoptotic to Viable Cells. J. Cell. Physiol..

[63] Schriek P., Villadangos J.A. (2023). Trogocytosis and cross-dressing in antigen presentation. Curr. Opin. Immunol..

[64] Drutman S.B., Trombetta E.S. (2010). Dendritic cells continue to capture and present antigens after maturation in vivo. J. Immunol..

[65] Lariccia V., Fine M., Magi S., Lin M.-J., Yaradanakul A., Llaguno M.C., Hilgemann D.W. (2011). Massive calcium–activated endocytosis without involvement of classical endocytic proteins. J. Gen. Physiol..

[66] Charpentier J.C., King P.D. (2021). Mechanisms and functions of endocytosis in T cells. Cell Commun. Signal..

[67] Doherty G.J., McMahon H.T. (2009). Mechanisms of Endocytosis. Annu. Rev. Biochem..

[68] Kaksonen M., Roux A. (2018). Mechanisms of clathrin-mediated endocytosis. Nat. Rev. Mol. Cell Biol..

[69] DOMIN J., PAGES F., VOLINIA S., RITTENHOUSE S.E., ZVELEBIL M.J., STEIN R.C., WATERFIELD M.D. (1997). Cloning of a human phosphoinositide 3-kinase with a C2 domain that displays reduced sensitivity to the inhibitor wortmannin. Biochem. J..

[70] Bouteau A., Kervevan J., Su Q., Zurawski S.M., Contreras V., Dereuddre-Bosquet N., Le Grand R., Zurawski G., Cardinaud S., Levy Y., Igyártó B.Z. (2019). DC Subsets Regulate Humoral Immune Responses by Supporting the Differentiation of Distinct Tfh Cells. Front. Immunol..

[71] Kaplan D.H., Li M.O., Jenison M.C., Shlomchik W.D., Flavell R.A., Shlomchik M.J. (2007). Autocrine/paracrine TGFbeta1 is required for the development of epidermal Langerhans cells. J. Exp. Med..

[72] Kashem S.W., Kaplan D.H. (2018). Isolation of Murine Skin Resident and Migratory Dendritic Cells via Enzymatic Digestion. Curr. Protoc. Immunol..

