# Supplementary material for: Dendritic cells overcome Cre/Lox induced gene deficiency by siphoning cytosolic material from surrounding cells
Source: iScience. 2024 Feb 6;27(3):109119. doi: 10.1016/j.isci.2024.109119 (PMC10879714; doi:10.1016/j.isci.2024.109119)
Supplement: Document S1. Figures S1‒S6 [file mmc1.pdf]

iScience, Volume *27*

Supplemental information

Dendritic cells overcome Cre/Lox induced gene deficiency by siphoning cytosolic material from surrounding cells

Christopher H. Herbst, Aurélie Bouteau, Evelin J. Menykő, Zhen Qin, Ervin Gyenge, Qingtai Su, Vincent Cooper, Neil A. Mabbott, and Botond Z. Igyártó

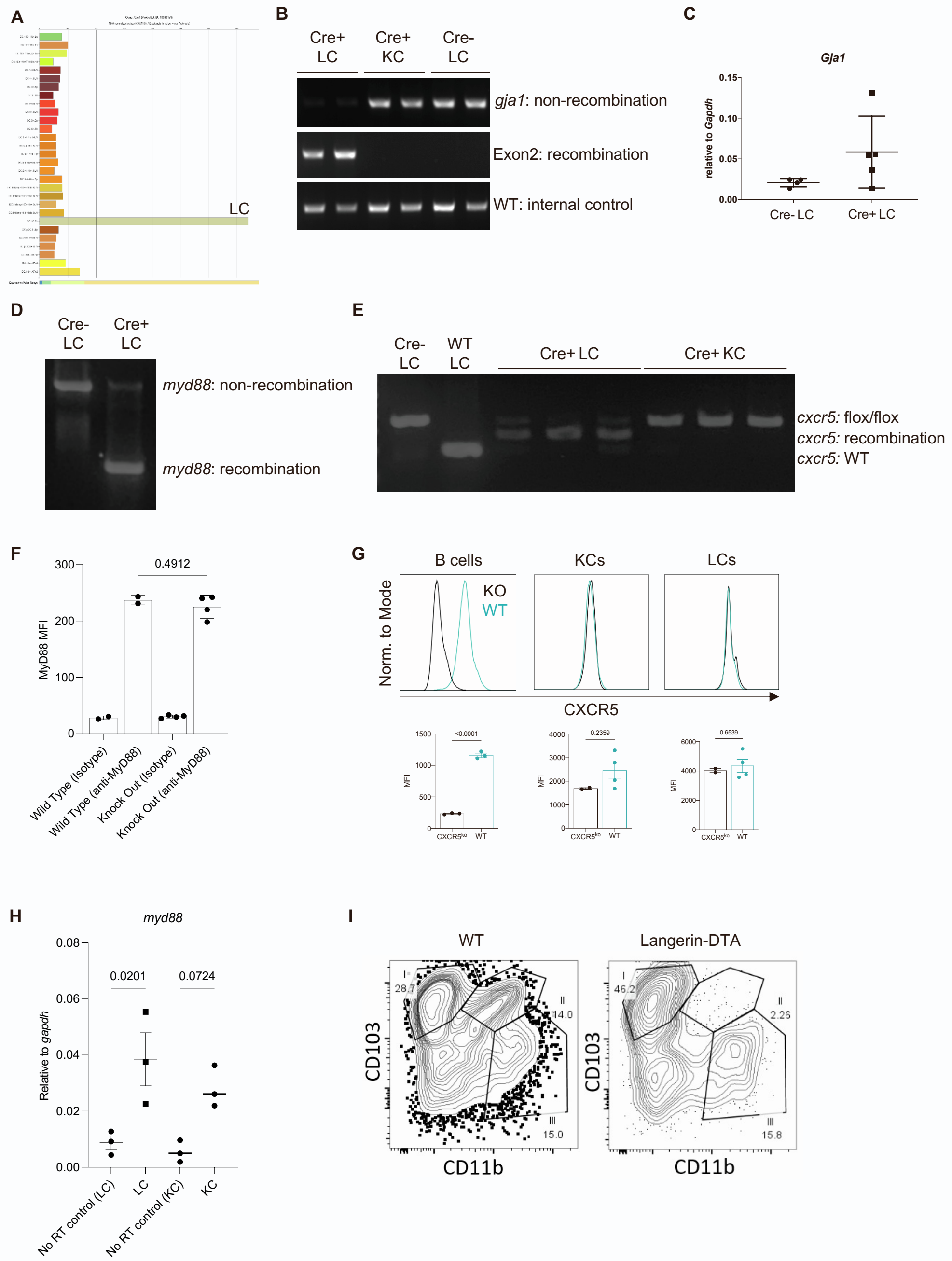

Figure S1. DCs can overcome gene deficiency, related to Figure 1. (**A**) Results of a *gja1* (Cx43) query into the ImmGen microarray database showing levels of *gja1* RNA transcripts in dendritic cell subsets. (**B**) Successful genetic recombination of *gja1* (Cx43*)* locus in Cre+ LCs, but not KCs or Cre- LCs. Cells were sorted from the epidermis of Cre positive and negative mice. (**C**) *gja1* mRNA levels in LCs sorted from Cre negative and positive mice. Each dot represents a separate mouse. One representative experiment of two shown. (**D**) Successful genetic recombination of *myd88* locus in Cre+ LCs, but not Cre- LCs. Cells were sorted from the epidermis of Cre positive and negative mice. (**E**) Successful genetic recombination of *cxcr5* locus in LCs sorted from Cre+ mice. (**F**) Intracellular MyD88 flow staining of whole blood from wild type or MyD88 global knock out mice compared to isotype control. Dots represent individual mice. Results from a single experiment. (**G**) CXCR5 staining of B cells, KCs, or LCs from CXCR5 global knock out mice or wild type mice and summary graphs. One representative experiment of two shown. (**H**) *myd88* mRNA levels relative to housekeeping gene *gapdh* in LCs and KCs sorted from wild type mice. Results from a single experiment. (**I**) Example flow plots showing CD11b+CD103+ mesenteric lymph node DCs (**II**) and their deletion in a Langerin-DTA mouse.

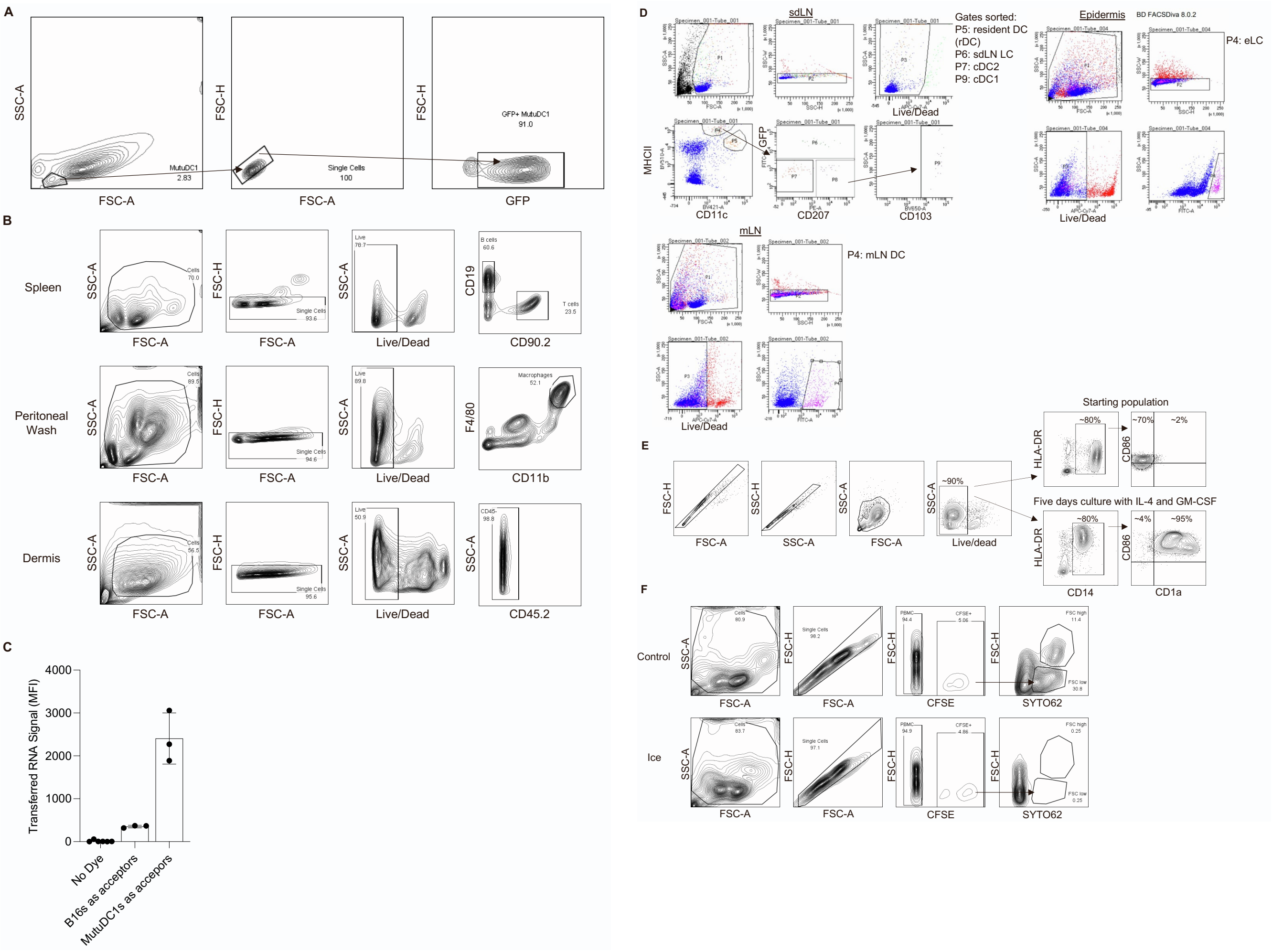

Figure S2. Intracellular material acquisition from surrounding cells is specific to DCs and universal among all DC subsets tested, related to Figure 2. (**A**) Gating strategy for distinguishing GFP+ or CFSE positive cells after co-culture. (**B**) Gating strategy for sorting splenic T and B cells, peritoneal macrophages, and dermal CD45- cells. (**C**) Comparison of the transferred RNA signal contained within B16s or DCs. Either B16s or DCs were RNA labeled then co-cultured with the other cell type for 45 minutes at a 1:1 ratio. Dots represent individual replicates. One of two replicate experiments shown. (**D**) Gating strategy for sorting epidermal Langerhans cells, skin draining lymph node migratory Langerhans cells, sdLN cDC1, cDC2, and resident DCs, and mesenteric lymph node migratory DC from Cre+ hLangCre-YFPf/f mice. (**E**) Differentiation of human DCs from blood monocytes gating strategy. (**F**) Gating strategy for identifying CFSE labeled DCs in DC/PBMC co-cultures. Summary plots calculated with FSC-H high populations.

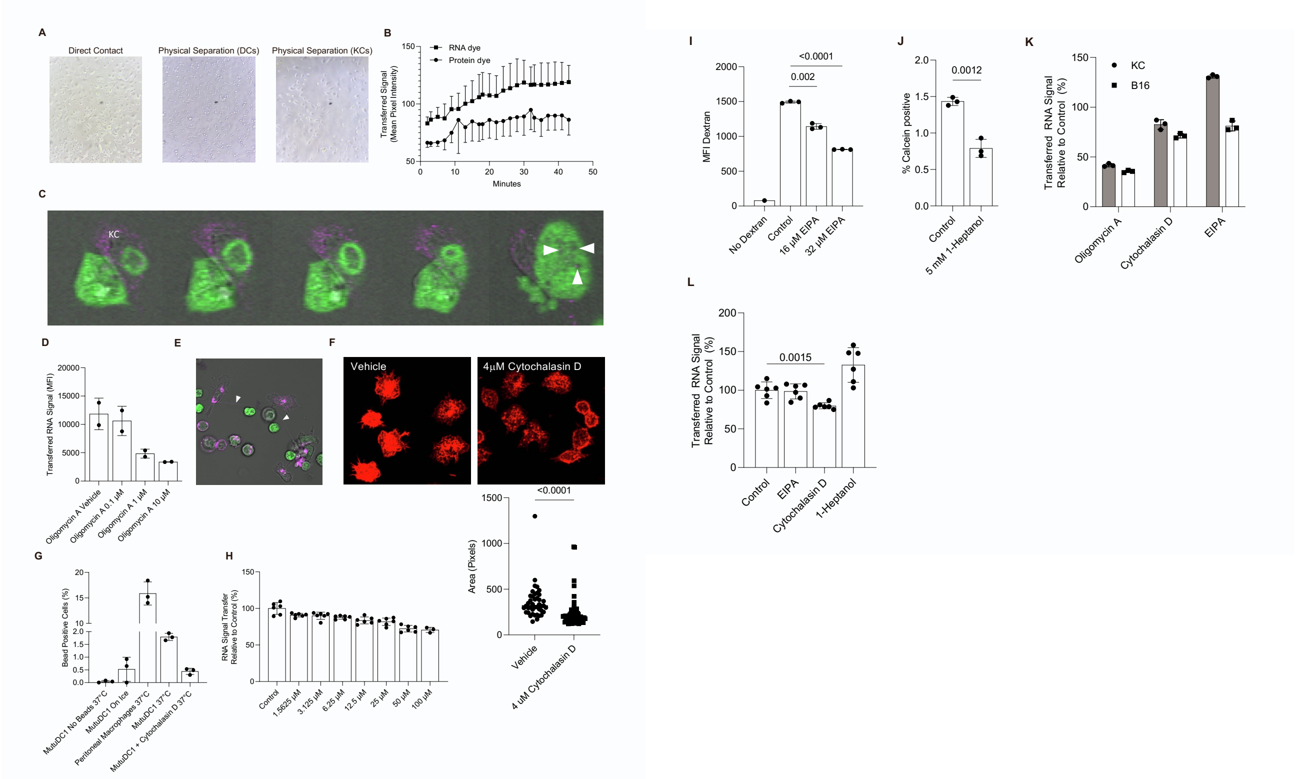

Figure S3. Dendritic cells siphon RNA from neighboring cells through a contact dependent mechanism that does not resemble conventional means of antigen uptake, related to Figure 3. (**A**) Representative images of: MutuDC1/keratinocyte direct co-culture, MutuDC1 cells on the bottom of the well, or keratinocytes adherent to the 8 mm coverslip while suspended overtop of MutuDC1s. (**B**) Mean pixel intensity of transferred RNA or CTV signals contained within areas identified to be dendritic cells over a 45 minute live cell time lapse. Each point represents the average of 4 fields of view of RNA or CTV signal contained within DC areas. Results from a single experiment shown. (**C**) Confocal single Z-plane slice of MutuDC1 cell (green) interacting with SYTO62 stained keratinocyte (purple). White arrowheads point to RNA containing vesicles in DCs. (**D**) Transferred RNA signal contained within MutuDC1 cells after a 45 minute co-culture with RNA labeled KCs containing indicated concentrations of Oligomycin A. One of three repeat experiments shown. (**E**) Example of TnTs (white arrowheads) forming between DCs (purple) in DC/KC co-cultures. (**F**) Rhodamine-Phalloidin staining of MutuDC1s in the presence of absence of 4 μM Cytochalasin D. Cell area measurements of individual cells in the presence or absence of 4 μM Cytochalasin D. Dots represent individual cells from a single experiment. (**G**) Percentage of MutuDC1 or peritoneal macrophages that have phagocytosed at least one 2μm microsphere in the presence or absence of cytochalasin D. One representative experiment of two shown. (**H**) Transferred RNA signal contained within MutuDC1 cells after a 45 minute incubation with RNA labeled B16 donor cells in the presence of the indicated concentration of cytochalasin D. Data combined and normalized from two experiments (**I**) MutuDC1 TRITC mean fluorescence intensity after a 30 minute incubation in media containing 10 kD TRITC Dextran and the indicated concentration of EIPA or vehicle control. One representative experiment of two shown. (**J**) Percent Calcein positive acceptor KCs (CTV labeled) in the presence or absence of 5 mM 1-heptanol. One representative experiment of two shown. (**K**) Transferred RNA signal contained within MutuDC1 cells after a 45 minute co-culture with either RNA labeled KCs or B16s in media containing 1 μM Oligomycin A, 8 μM Cytochalasin D, or 32 μM EIPA. One representative experiment of two shown. (**L**) Transferred RNA signal in MHCII+ B220- F4/80- spleen cells after a 45 minute co-culture with RNA labeled B16 cells in the presence or absence of 32 μM EIPA, 8 μM cytochalasin D, or 5mM 1-Heptanol. Data normalized and combined from two separate experiments.

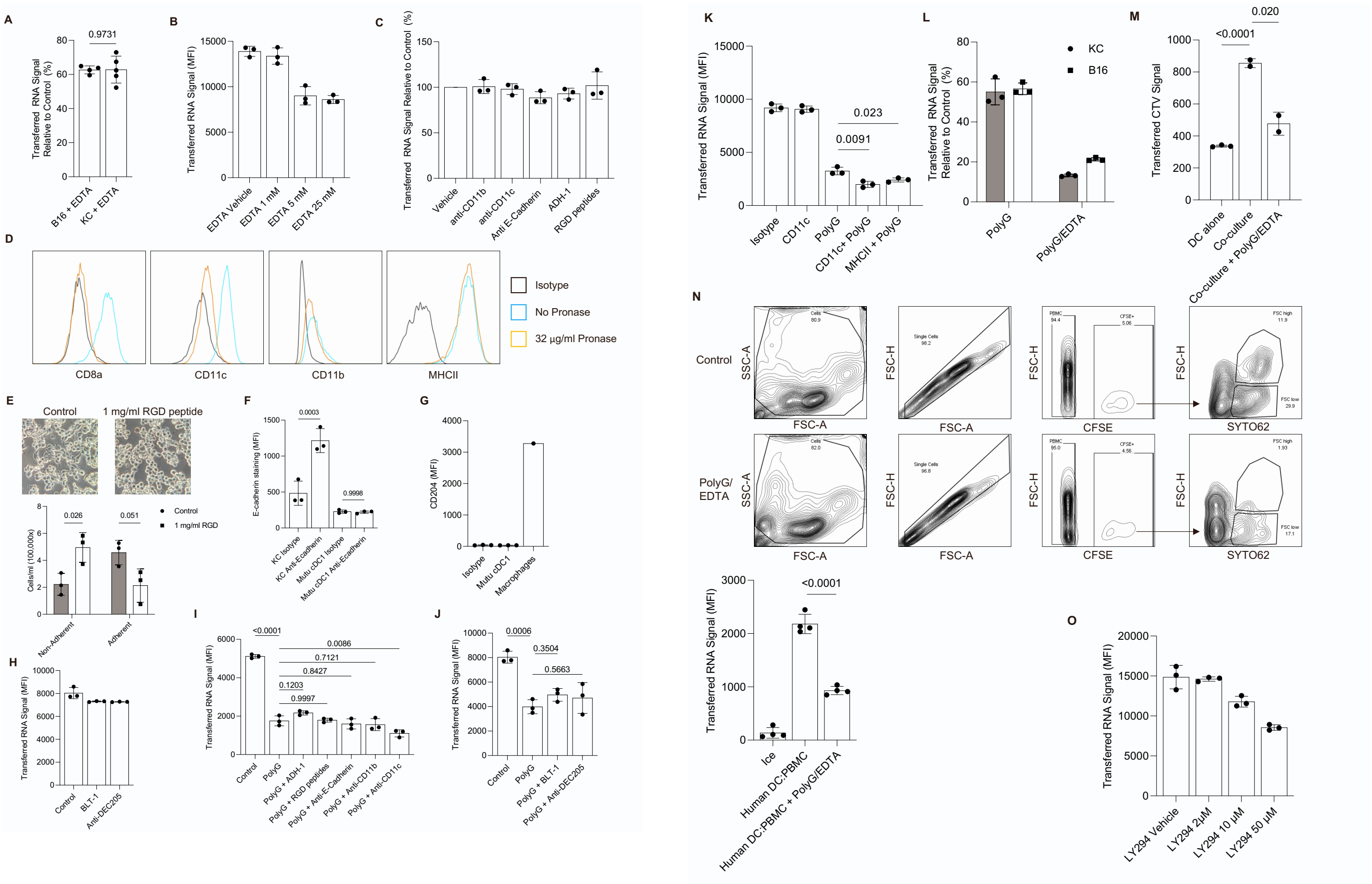

Figure S4. RNA transfer is dependent on calcium and can be partially blocked with the scavenger receptor inhibitor Polyguanylic acid, related to Figure 4. (**A**) Percent inhibition compared to control of RNA signal contained within MutuDC1s after a 45 minute co-culture with either RNA labeled keratinocytes or B16 cells in the presence of 5 mM EDTA. (**B**) RNA signal contained within MutuDC1 cells after a 45 minute co-culture with RNA labeled KC containing indicated concentrations of EDTA. (**C**) Percent inhibition compared to control of RNA signal contained within MutuDC1s after a 45 minute co-culture with B16s in the presence of 2.5 µg/ml anti-CD11b blocking antibody, 2.5 µg/ml anti-CD11c blocking antibody, 5 µg/ml anti-E-cadherin blocking antibody, 350 nM ADH-1, or 1mg/ml RGD peptide. (**D**) Staining of MutuDC1 cells or MutuDC1 cells treated with 32 µg/ml Pronase and antibodies against the indicated proteins at a concentration of 2.5 µg/ml. (**E**) Number of adherent and non-adherent B16 cells after a 45 minute incubation on collagen coated microscopy slides in the presence or absence of 1 mg/ml RGD peptide and images of cell morphology just prior to removal of supernatant. Results from a single experiment. (**F**) Binding of a blocking anti-E-cadherin antibody to either keratinocytes or MutuDC1s. Binding was detected using goat anti-rat IgG DyLight 550 conjugate antibody. Results from a single experiment. (**G**) MutuDC1 cells or peritoneal macrophages stained with 1 µg/ml anti-CD204 antibody. Results from a single experiment. (**H**) RNA signal contained within MutuDC1 after a 45 minute incubation with RNA labeled B16 cells in the presence of a 1µM SR-B1 inhibitor or 2.5 µg/ml DEC-205 blocking antibody. (**I, J**) RNA signal contained within MutuDC1 cells after a 45 minute co-culture with RNA labeled B16 cells in the presence of indicated inhibitors or antibodies plus 500 µg/ml PolyG. (**K**) RNA signal contained within MutuDC1 cells after a 45 minute co-culture with RNA labeled B16s in the presence of anti-CD11c or anti-MHC-II antibody + 500 µg/ml PolyG. (**L**) Transferred RNA signal relative to control contained within MutuDC1 cells after co-culture with either RNA labeled B16s or keratinocytes in the presence of 500 µg/ml PolyG or 500 µg/ml PolyG + 5 mM EDTA. (**M**) CTV signal measured within MutuDC1 cells after 4 hour co-culture with CTV labeled B16 cells in the presence or absence of 500 µg/ml PolyG + 5 mM EDTA. (**N**) Gating strategy for identifying CFSE labeled DCs in DC/PBMC co-cultures. Summary plots calculated with FSC-H high CFSE+ populations. One representative experiment out of two is shown. (**O**) RNA signal contained within MutuDC1 cells after a 45 minute co-culture with RNA labeled B16s containing indicated concentrations of the PI3K inhibitor LY294002. All experiments were repeated three times unless otherwise indicated.

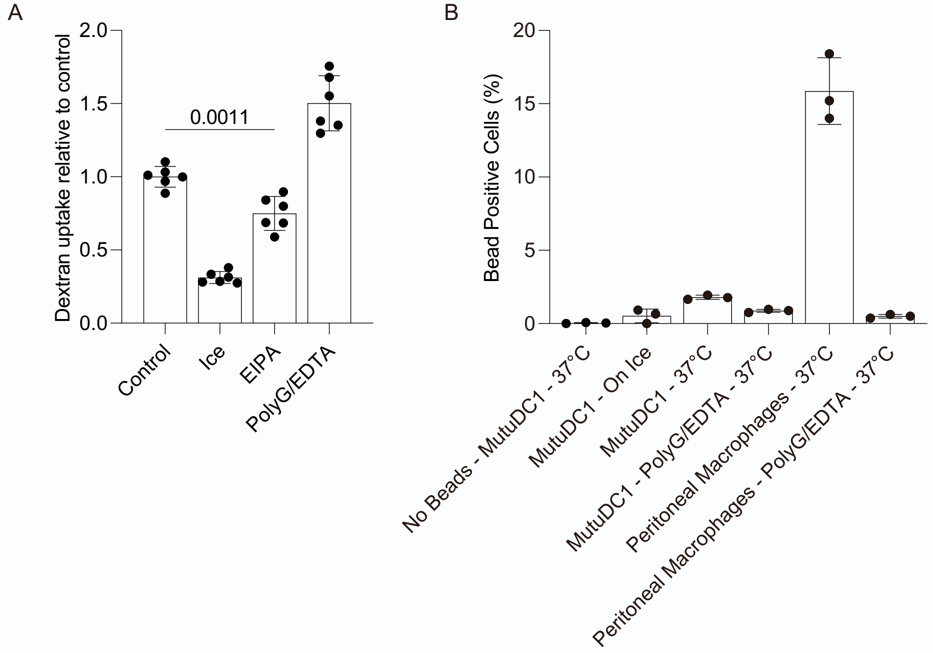

Figure S5. DCs present the antigen acquired through intracellular monitoring on both MHC-I and MHC-II, related to Figure 5. (**A**) TRITC-Dextran (10kD) signal in MutuDC1 cells after a 30 minute incubation in DC media containing 32 µM EIPA or 500 µg/ml PolyG + 5mM EDTA. Data normalized and combined from two separate experiments. (**B**) Percentage of MutuDC1 cells or peritoneal macrophages that phagocytosed at least one 2 µm microsphere in the presence or absence of 500 µg/ml PolyG + 5mM EDTA. One representative experiment of two shown.

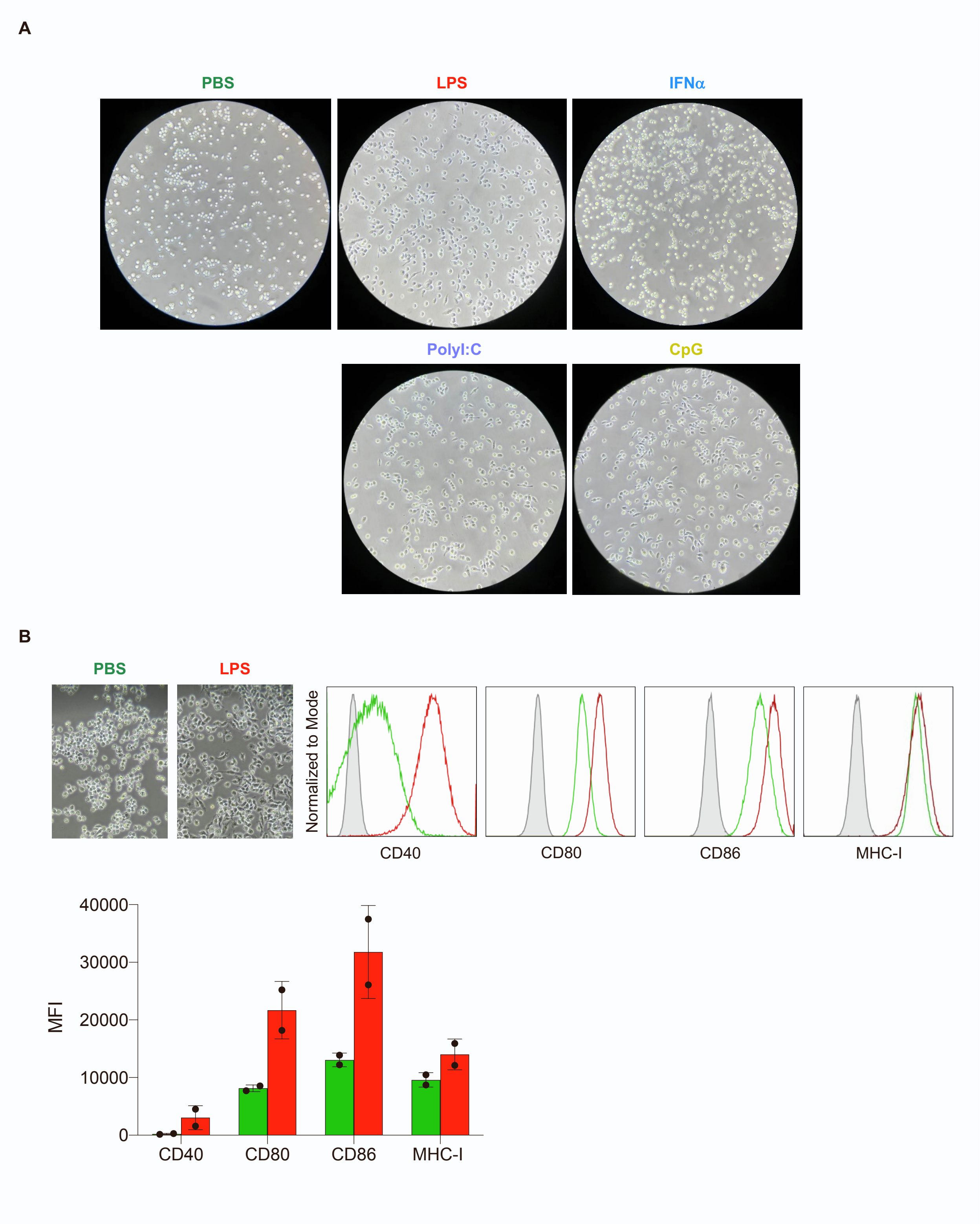

Figure S6. DC maturation induced by inflammatory signals do not affect intracellular monitoring, related to Figure 7. (**A**) Light microscope images of MutuDC2 cells after 12 hour incubation with 1 µg/ml LPS, 10 µg/ml INFα, 5 µg/ml Poly I:C, 0.5 µM CpG, or PBS control. (**B**) Co-stimulatory marker and MHC-I expression in MutuDC2 cells after 12 hour incubation with 1 µg/ml LPS. One representative experiment of two shown.
